# Bacterial fitness in chronic wounds appears to be mediated by the capacity for high-density growth, not virulence or biofilm functions

**DOI:** 10.1371/journal.ppat.1007511

**Published:** 2019-03-20

**Authors:** Sarah J. Morgan, Soyeon I. Lippman, Gilbert E. Bautista, Joe J. Harrison, Christopher L. Harding, Larry A. Gallagher, Ann-Chee Cheng, Richard Siehnel, Sumedha Ravishankar, Marcia L. Usui, John E. Olerud, Philip Fleckman, Randall D. Wolcott, Colin Manoil, Pradeep K. Singh

**Affiliations:** 1 Department of Microbiology, University of Washington, Seattle, WA, United States of America; 2 Department of Genome Sciences, University of Washington, Seattle, WA, United States of America; 3 Department of Biological Sciences, University of Calgary, Calgary, AB, Canada; 4 Department of Medicine, University of Washington, Seattle WA, United States of America; 5 Southwest Regional Wound Care Center, Lubbock, TX, United States of America; Children's Hospital Boston, UNITED STATES

## Abstract

While much is known about acute infection pathogenesis, the understanding of chronic infections has lagged. Here we sought to identify the genes and functions that mediate fitness of the pathogen *Pseudomonas aeruginosa* in chronic wound infections, and to better understand the selective environment in wounds. We found that clinical isolates from chronic human wounds were frequently defective in virulence functions and biofilm formation, and that many virulence and biofilm formation genes were not required for bacterial fitness in experimental mouse wounds. In contrast, genes involved in anaerobic growth, some metabolic and energy pathways, and membrane integrity were critical. Consistent with these findings, the fitness characteristics of some wound impaired-mutants could be represented by anaerobic, oxidative, and membrane-stress conditions *ex vivo*, and more comprehensively by high-density bacterial growth conditions, in the absence of a host. These data shed light on the bacterial functions needed in chronic wound infections, the nature of stresses applied to bacteria at chronic infection sites, and suggest therapeutic targets that might compromise wound infection pathogenesis.

## Author summary

Chronic infections are poorly understood, hard to model, and treatment resistant. Future progress depends upon understanding infection pathogenesis. Our study suggests that many bacterial virulence and biofilm formation functions are not required for fitness of infecting bacteria in chronic wounds. Instead, we found that functions that mitigate stresses associated with high-density bacterial growth are critical for infection. These findings suggest new approaches to model and target chronic bacterial infections.

## Introduction

The diversity of bacterial-host interactions is extraordinary and ranges from symbioses critical for health, to infections that rapidly kill. Even more remarkable is the fact that the same species of bacteria can produce markedly different disease manifestations, depending upon the clinical setting. The opportunistic pathogen *Pseudomonas aeruginosa* is a prime example. In some patients, like those with pneumonia or sepsis, it produces acute infections that can be rapidly lethal [1]. In other patients, like those with chronic wounds, sinus disease, or cystic fibrosis (CF), large numbers of organisms can remain localized at infection sites for years without systemic spread [1]. *Staphylococcus aureus*, *Escherichia coli*, *Streptococcus sp*. and others also produce life-threatening acute infections in some settings and smoldering chronic infections in others [2].

While knowledge of acute infection pathogenesis has advanced, understanding of chronic infections has lagged. An emerging model suggests chronic infection may involve a two-step process, with different functions mediating initial and established phases of infection [3–5]. Early-on, acute virulence functions are postulated to be active, enabling bacteria to establish a foothold within the host [3, 4]. Once infection is established, virulence factor activity may diminish, and biofilm formation is induced [3–5]. Biofilms are surface-attached and matrix-encased communities that are resistant to killing by antibiotics and immune responses. The net effect of these mechanisms could enable infection initiation, but yet produce the non-invasive and persistent phenotype of chronic infections.

While this model is compelling, the evidence supporting phased activity of virulence and biofilm formation is inconclusive. For example, work using a murine surgical wound model found many genes which influence virulence such as motility, LPS, and quorum sensing were not essential for survival in chronic wound [6]. The only virulence factor which was both upregulated and important for fitness in this chronic wound model was the type 3 secretion system [6]. Studies of CF isolates has shown that bacterial genes mediating acute virulence are frequently inactivated by mutations acquired as infecting bacteria evolve *in vivo* [3, 7, 8]. This finding is consistent with the idea that some virulence factors could be dispensable, and indeed disadvantageous for infecting bacteria once chronic infection is established. However, it does not provide information about their activity during the initiation or early phases of infection.

The importance of biofilm formation functions in chronic infections is also unsettled. For example, examination of infected specimens suggests that many bacteria in infected wounds and CF lungs are living in biofilm-like bacterial aggregates [9–11]. Furthermore, biofilm-mediated antibiotic tolerance is consistent with the treatment-resistant phenotype of chronic infections [11–13]. However, isolates taken from chronic CF infections often exhibit *in vitro* biofilm defects [14–16], and genes encoding a key biofilm-mediated polysaccharide was found to be repressed in murine chronic wounds [6]. In addition, recent work indicates that environmental conditions at infection sites (such as viscous mucus [17], proteases that inactivate flagella [17], and /or polymer-rich secretions[18]) can force bacteria to grow as aggregates. Thus, it is not known if aggregates form *in vivo* via the activity of biofilm functions, or if host conditions are responsible.

Here we performed studies to test the importance of virulence and biofilm formation functions in wounds, and better understand wound infection pathogenesis. We studied human wound infection isolates, and performed genomic-level analysis in *P*. *aeruginosa* to identify bacterial functions that mediate bacterial fitness in murine chronic wounds. We also attempted to model the selective environment in wounds using *ex vivo* conditions. We focused on chronic wounds as they are a major and understudied problem, and *P*. *aeruginosa* is a predominant wound pathogen.

## Results

### Several virulence and biofilm functions are often inactive in wound isolates

Studies with CF *P*. *aeruginosa* isolates indicate that virulence and biofilm formation functions are frequently inactivated during within-host evolution [3, 7, 19, 20]. To determine if wound isolates exhibited similar phenotypes, we studied isolates from seven chronic wounds. We tested 51–480 *P*. *aeruginosa* isolates per wound to account for diversity that may exist within the population. Because of the large number of isolates tested, we focused on phenotypes that could be tested with high throughput.

In contrast to *P*. *aeruginosa* isolates from environmental sources and acute infections, many wound isolates exhibited virulence factor defects and were biofilm-deficient (Table 1, S1 Fig). Most wounds were dominated by isolates defective in protease and rhamnolipid (a surfactant toxin) production, and twitching motility. In addition, ~one-quarter of isolates were incapable of swimming and ~one-quarter exhibited marked defects in a standard biofilm assay [21]. Notably, the motility and biofilm formation phenotypes of isolates were often discordant (S2 Fig).

**Table 1:** Virulence and biofilm functions are often inactive in wound isolates.

Isolate Source	% Protease Negative (# negative/# total)		% Rhamnolipid Negative (# negative/# total)		% Twitching Motility Negative (# negative/# total)		% Biofilm Deficient (#negative/#total)		% Swimming Motility Negative (# negative/# total)	
Wound #1	67%	(306/458)	69%	(314/457)	98%	(450/457)	69%	(43/62)	65%	(298/458)
Wound #2	0%	(0/480)	9%	(42/472)	97%	(466/480)	67%	(34/51)	0%	(2/480)
Wound #3	58%	(90/154)	100%	(154/154)	95%	(146/154)	9%	(5/57)	0%	(0/154)
Wound #4	0%	(0/96)	38%	(36/96)	100%	(95/95)	3%	(2/59)	0%	(0/96)
Wound #5	100%	(87/87)	100%	(87/87)	100%	(87/87)	0%	(0/61)	61%	(53/87)
Wound #6	1%	(1/93)	87%	(81/93)	88%	(82/93)	14%	(8/57)	0%	(0/93)
Wound #7	100%	(190/190)	100%	(190/190)	100%	(190/190)	0%	(0/59)	1%	(1/190)
**Total Wound**	**43%**	**(674/1558)**	**58%**	**(904/1549)**	**97%**	**(1516/1556)**	**22%**	**(101/455)**	**23%**	**(354/1558)**
Blood	33%	(2/6)	33%	(2/6)	17%	(1/6)	33%	(2/6)	33%	(2/6)
Neonatal	10%	(1/10)	22%	(2/9)	10%	(1/10)	0%	(0/10)	0%	(0/10)
Pneumonia	0%	(0/20)	5%	(1/20)	5%	(1/20)	0%	(0/20)	15%	(3/20)
**Total Acute**	**8%**	(3/36)*	**14%**	(5/36)*	**8%**	(3/36)*	**6%**	**(2/36)**	**14%**	**(5/36)**
**Environment**	**0%**	(0/14)*	**8%**	(1/14)*	**0%**	(0/14)*	**0%**	**(0/14)**	**0%**	**(0/14)**

* significantly different (p<0.5) by Fisher exact test. For statistical analysis each wound was determined to be positive or negative for each test based on the phenotype of the majority of isolates.

In many cases wound *P*. *aeruginosa* populations contained both isolates that did and did not produce the tested factor, thus it is possible that expression by a small subpopulation is sufficient for infection. However, all sampled isolates in two wounds (87 isolates in wound 5 and 190 isolates in wound 7) lacked protease, rhamnolipids or twitching motility, raising the possibility that these factors may be completely dispensable in some chronic wounds.

### Modification of a chronic wound model to study infection initiation

The virulence and biofilm formation defects of human wound isolates could be explained if these functions were required for infection initiation but not necessary for fitness later in infection, or if they were not necessary at any stage of pathogenesis. We used a wound model developed in diabetic mice [9, 22] to distinguish between these possibilities.

In this model, punch biopsy wounds are created on the backs of diabetic (Db/Db) mice, inoculated with *P*. *aeruginosa* two days later, and then covered with a semi-occlusive dressing. The resulting infection lasts for over six-weeks [9, 22]. The model was originally developed using biofilm-grown bacteria as the inoculum [9, 22].We modified the model to use liquid-culture (free-living) *P*. *aeruginosa* as the inoculum so that infection defects could be detected if biofilm formation functions were important in this model.

**Fig 1:**
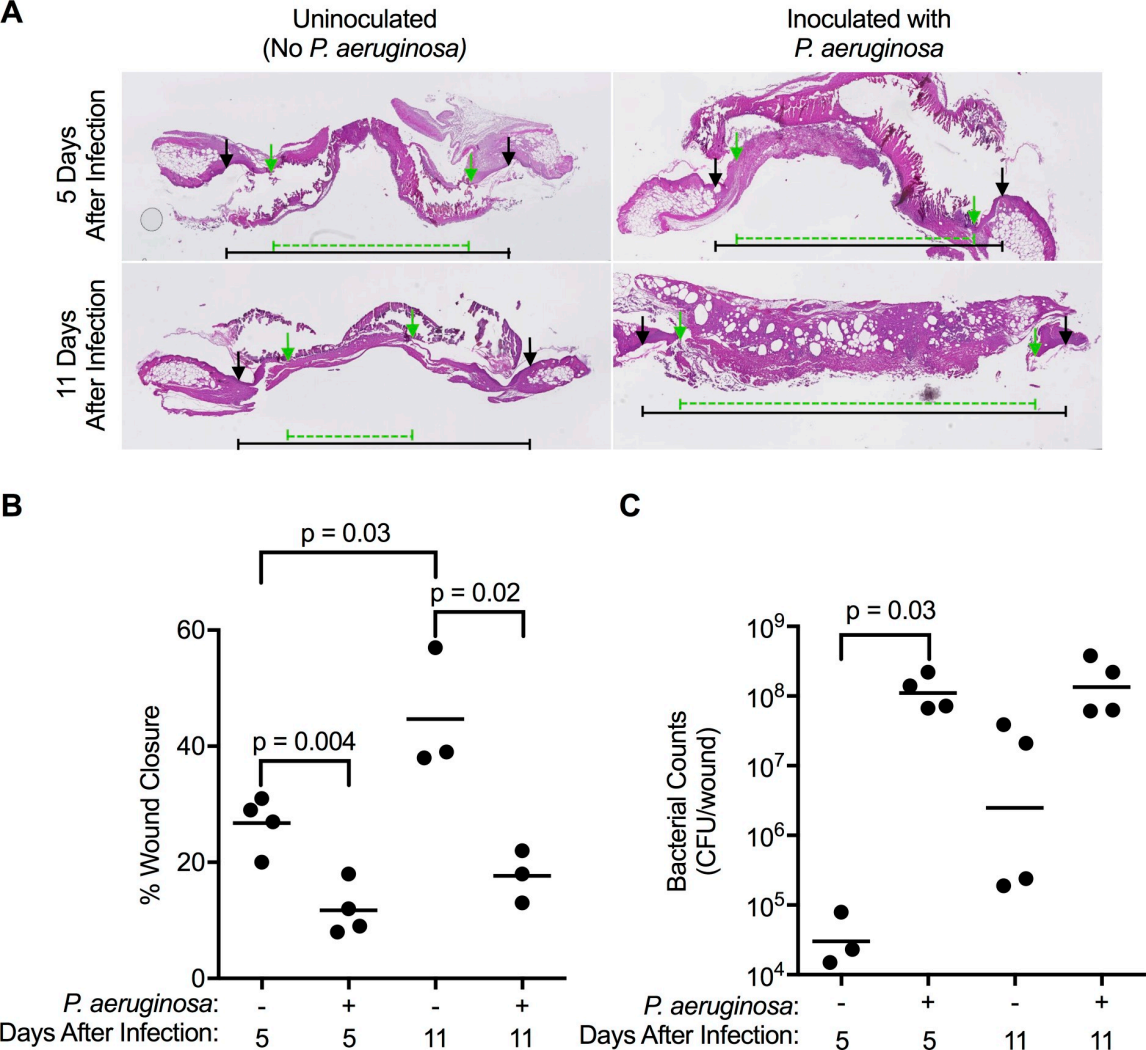
Wound infections initiated with *P*. *aeruginosa* liquid cultures delay wound healing. **A.** H&E staining of excised wounds show original wound edges (black arrows) and the extent of epithelial migration after 5 or 11 days of healing (green arrows). **B.** The extent of wound closure (healing) was delayed in wounds challenged with *P*. *aeruginosa*. **C.** Total CFU recovered from wounds shows *P*. *aeruginosa*-challenged wounds contained high bacterial densities, and unchallenged wounds harbored low densities of bacteria (likely due to spontaneous infection *Staphlococcus sp*. as previously observed [52]). For A and B, each point represents data from an individual mouse. Significance determined by Mann-Whitney analysis.

Similar to results with pre-grown biofilms [9, 22], the liquid culture inoculum established infection, delayed wound healing (Fig 1A and 1B), and bacteria persisted for the length of the study (experiments were terminated after 11 days) (Fig 1C) without producing systemic illness (S3 Fig). Because our objective was to identify genetic requirements for infection initiation we assayed wound bacteria after five or seven days in subsequent experiments.

### Inactivation of type 3 secretions compromised wound fitness, but other factors did not

**Fig 2:**
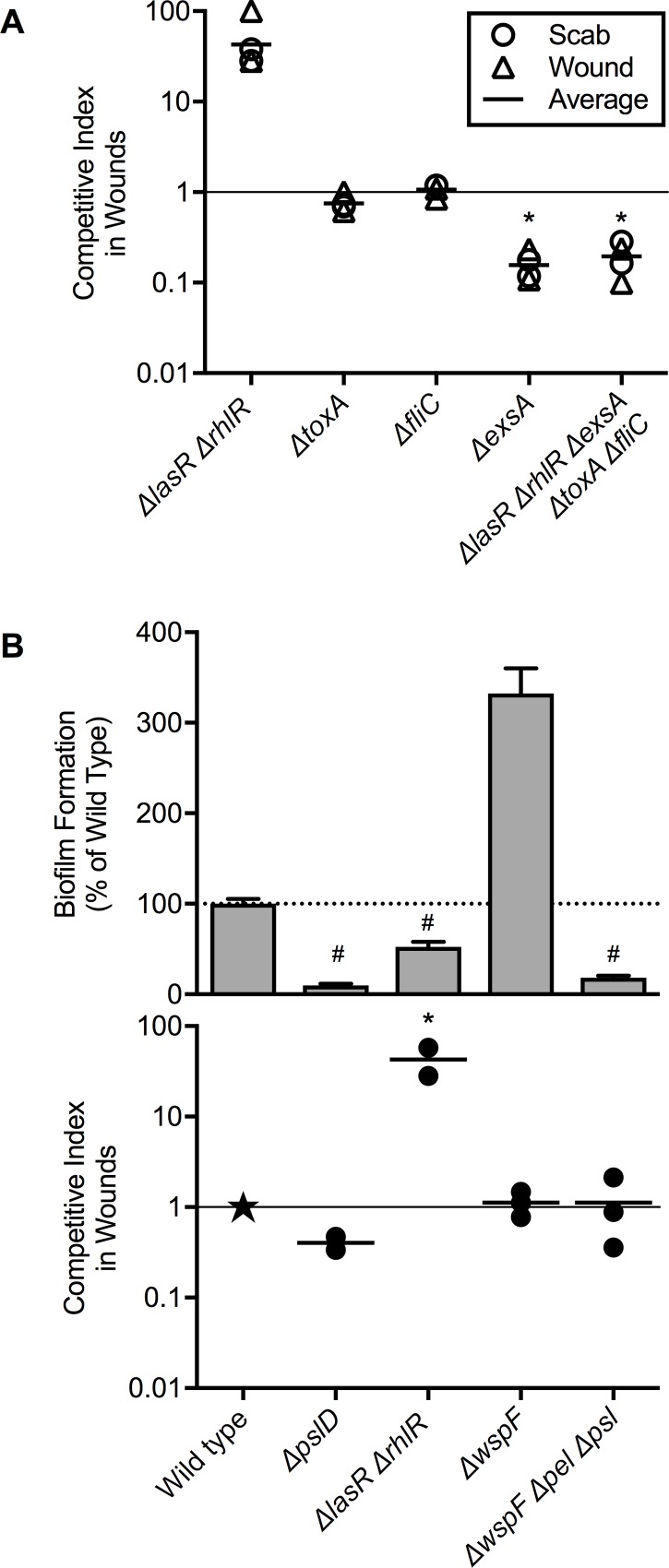
Loss of acute virulence and biofilm formation functions does not decrease fitness in chronic wound infections. **A.** Mutants lacking indicated acute virulence genes were competed 1:1 against wild-type *P*. *aeruginosa* in murine wounds, and competitive index was measured in scabs (circles) and wound beds (triangles). Line indicates mean values. **B.** Mutants defective and enhanced in biofilm formation (top) were competed 1:1 against wild-type *P*. *aeruginosa* in murine wounds (bottom). Scab and wound bed were harvested together (filled circle). Wild-type *P*. *aeruginosa* was assigned a competitive index of 1 (star). * indicates competitive index significantly different than 1 (p<0.01) by one sample t test. ^#^ indicates significant difference from wild type (p<0.05) by one-way ANOVA followed by Dunnett’s test.

We began testing the effect of virulence factors on wound fitness by comparing wild type *P*. *aeruginosa* and an otherwise isogenic mutant lacking both *las* and *rhl* quorum-sensing systems. Quorum sensing controls key virulence factors including rhamnolipids, proteases, pyocyanin, and cyanide [23]. Despite lacking expression of these factors, the quorum-sensing mutant (Δ *lasR* Δ *rhlR*) exhibited a 40-fold advantage over wild-type *P*. *aeruginosa* in 1:1 competitions (Fig 2A), similar to observations in other models [24].

Because wounds sites can differ regionally in nutrient and oxygen availability and inflammation [9, 22, 25], we separately measured fitness in the exudate above wounds (the “scab”) and the wound bed, but found minimal differences (Fig 2A). Furthermore, the Δ *lasR* Δ *rhlR* mutant exhibited no infection defect when tested in pure culture (S4 Fig). Thus, the mutant’s fitness advantage in competition is not likely due to a “cheating” mechanism [26] wherein the mutant exploits quorum-controlled factors produced by wild-type bacteria.

We also tested mutants defective in exotoxin A and flagellum (Δ*fliC)* and found no fitness defects relative to wild-type bacteria in the scab or wound bed (Fig 2A). The only virulence function tested affecting wound fitness was the type 3 secretion system (Δ*exsA*), which produced a six-fold defect (Fig 2A) when inactivated (p<0.001). This finding is consistent with previous work indicating that *P*. *aeruginosa* induces Type 3 secretion during wound infection [6]. Furthermore, a mutant lacking all these virulence functions (Δ*lasR*, Δ*rhlR*, Δ*toxA*, Δ*fliC*, Δ*exsA*,) had only a 5-fold defect in wound fitness (Fig 2A), similar to that of Δ*exsA*.

### Biofilm formation functions do not affect wound fitness

We tested isogenic mutants with diminished and enhanced *in vitro* biofilm formation phenotypes in the murine wound model, and found no relationship between the mutants’ capacity to form biofilms and wound fitness. For example, inactivating a primary biofilm matrix polysaccharide (Δ*pslD*) or quorum-sensing (Δ*lasR* Δ*rhlR*) produced biofilm defects, but no wound defect (Fig 2B). Likewise, inactivating the *wspF* negative regulator markedly increased biofilm formation (likely by raising levels of ci-di-GMP [27]), but did not increase wound fitness (Fig 2B). Finally, inactivating the production of pel and psl extracellular polysaccharides in the Δ*wspF* background decreased biofilm formation (compared to Δ*wspF)*, but wound fitness was unchanged (Fig 2B). Thus, genetic manipulations that markedly altered biofilm formation did not produce corresponding changes in bacterial wound fitness, or change the apparent clinical manifestations of infection in mice.

### Genomic screen to identify bacterial functions needed in chronic wounds

If biofilm formation and virulence functions do not generally affect bacterial fitness in chronic wounds, what functions do? We addressed this using Tn-seq experiments [28]. Tn-seq enables the fitness of transposon mutants in nearly every non-essential gene to be measured simultaneously. Tn-seq measures fitness by enumerating the relative abundance of each transposon mutant before and after selection (in wounds in this case) by sequencing transposon-genome junctions, which serve as markers of each mutant [28].

**Fig 3:**
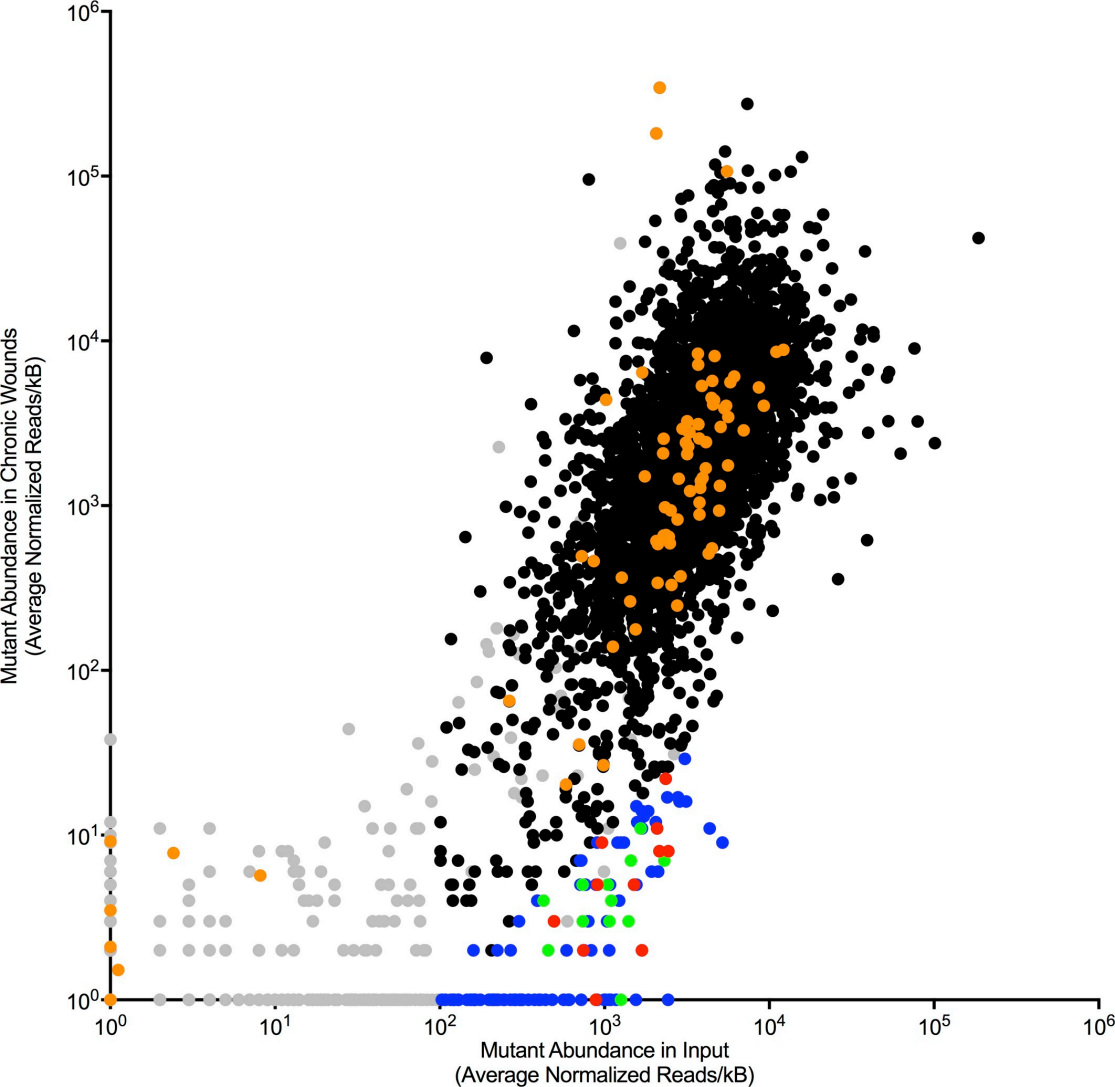
Identification of gene inactivations that compromise wound fitness. Tn-seq measured the relative change in abundance of mutants in the inoculum (x-axis) and after infection (y-axis). Each point represents a different gene inactivation. Gray points indicate gene inactivations with low input abundance; orange points indicate virulence genes [29]; blue, green and red points indicate gene inactivations exhibiting significant (P<0.01) decreases in abundance after infection. Green and red points indicate gene inactivations tested in 1:1 experiments (see Fig 4); gene inactivations indicated by green points were verified to be wound-defective, while gene inactivations indicated by red points were not defective in verification experiments. Gene inactivations indicated by blue points were not tested in 1:1 competitions.

The input pool we used contained ~ 20 mutants in each of ~ 5500 genes in *P*. *aeruginosa* (PA01) for a total of ~110,000 mutants. We excluded 567 genes that had low representation in the input pool from further analysis (Fig 3, gray points; S1 Table). Genes were excluded from further analysis if there were less than 100 reads/kb, or only a single transposon insertion in that gene in the input sample.

Mutants in 28 genes were completely eliminated after infection in all 4 independent infection experiments performed, and mutants in an additional 114 genes exhibited significant (p<0.01) fitness defects in wounds (Fig 3, blue points; Table 2 and S1 Table). Notably, 11 of 28 gene mutations that were eliminated during infection were genes of unknown function (S1 Table). Consistent with our finding that virulence functions were often inactivated in *P*. *aeruginosa* isolated from human wounds (Table 1), no gene inactivations linked to host toxicity or invasion [29] exhibited significant fitness defects in the murine wound model (virulence genes [29] were indicated by orange points in Fig 3).

**Table 2:** Genes with decreased representation following Tn-seq indicating a possible role of these genes in fitness in the chronic wound infection.

Locus	Gene	Average normalized reads/kb*		Selection Ratio**	Fitness in 1:1 competition***
		Inoculum	Wounds		
PA3351	flgM	2429	0	0.000	<0.01
PA0984	-	1090	0	0.000	0.3±0.1
PA0597	-	997	0	0.000	nt
PA5070	tatC	904	0	0.000	8.2±0.9
PA3807	ndk	723	0	0.000	nt
PA4951	orn	610	0	0.000	<0.01
PA3743	trmD	437	0	0.000	nt
PA4530	-	407	0	0.000	nt
PA3480	-	399	0	0.000	nt
PA1013	purC	345	0	0.000	nt
PA5049	rpmE	317	0	0.000	nt
PA0906	-	314	0	0.000	nt
PA5365	phoU	308	0	0.000	nt
PA3647	-	305	0	0.000	nt
PA1548	-	281	0	0.000	nt
PA3244	minD	247	0	0.000	nt
PA1742	-	215	0	0.000	nt
PA1480	ccmF	207	0	0.000	nt
PA2491	-	206	0	0.000	nt
PA4945	miaA	199	0	0.000	0.02±0.01
PA3721	nalC	176	0	0.000	nt
PA1929	-	165	0	0.000	nt
PA3263	-	162	0	0.000	nt
PA3998	-	162	0	0.000	nt
PA0025	aroE	160	0	0.000	nt
PA1479	ccmE	146	0	0.000	nt
PA4600	nfxB	129	0	0.000	nt
PA5288	glnK	122	0	0.000	nt
PA5322	algC	602	0.3	0.000	0.04±0.01
PA2876	pyrF	1176	0.5	0.000	nt
PA1547	-	1014	0.5	0.001	nt
PA4723	dksA	1551	0.8	0.001	nt
PA0943	-	1258	0.9	0.001	<0.01
PA3243	minC	566	0.5	0.001	nt
PA4431	-	1069	1.1	0.001	nt
PA1057	-	481	0.6	0.001	nt
PA4430	-	375	0.5	0.001	nt
PA4551	pilV	1689	2.4	0.001	0.6±0.3
PA4732	pgi	886	1.3	0.002	0.70±0.07
PA4758	carA	5198	9.1	0.002	nt
PA5332	crc	1067	2.0	0.002	nt
PA2236	pslF	287	0.6	0.002	nt
PA5567	-	227	0.5	0.002	nt
PA2644	nuoI	829	1.7	0.002	nt
PA3108	purF	1400	3.0	0.002	<0.01
PA3005	nagZ	743	1.6	0.002	17±2
PA4855	purD	752	1.8	0.002	nt
PA5198	-	4342	10.5	0.002	nt
PA1766	-	130	0.3	0.002	nt
PA4553	pilX	1036	2.7	0.003	nt
PA5563	soj	153	0.4	0.003	nt
PA0945	purM	2115	5.6	0.003	nt
PA2852	-	103	0.3	0.003	<0.01
PA4834	-	1073	2.9	0.003	0.13±0.08
PA2637	nuoA	2310	6.9	0.003	0.13±0.08
PA3242	-	1514	4.6	0.003	16.58±0.00
PA2658	-	2443	7.5	0.003	10.9±2
PA5489	dsbA	1563	5.0	0.003	nt
PA0762	algU	1930	6.3	0.003	nt
PA2238	pslH	1524	5.2	0.003	nt
PA0966	ruvA	1509	5.2	0.003	nt
PA0666	-	1098	3.8	0.003	0.2±0.1
PA1476	ccmB	155	0.5	0.003	nt
PA1064	-	2155	7.6	0.004	8.4±0.3
PA0965	ruvC	1226	4.4	0.004	nt
PA3201	-	288	1.1	0.004	nt
PA5038	aroB	739	2.8	0.004	0.02±0.01
PA4283	recD	798	3.1	0.004	nt
PA0967	ruvB	212	0.9	0.004	nt
PA0760	-	588	2.5	0.004	nt
PA2965	fabF1	222	1.0	0.005	nt
PA0552	pgk	1446	6.7	0.005	<0.01
PA0944	purN	3125	15.7	0.005	nt
PA3338	-	1078	5.4	0.005	nt
PA2656	-	1047	5.3	0.005	0.5±0.1
PA5131	pgm	268	1.4	0.005	nt
PA3173	-	2087	11.1	0.005	1.0±0.7
PA4854	purH	243	1.3	0.005	nt
PA2648	nuoM	455	2.5	0.005	0.04±0.01
PA1180	phoQ	906	5.0	0.006	22 ±4
PA0401	-	2855	15.9	0.006	nt
PA4765	omlA	121	0.7	0.006	nt
PA4453	-	2045	11.7	0.006	nt
PA2241	pslK	889	5.2	0.006	nt
PA2398	fpvA	2796	17.2	0.006	nt
PA4462	rpoN	108	0.7	0.006	nt
PA4856	retS	715	4.6	0.006	nt
PA5425	purK	767	5.1	0.007	nt
PA1550	-	1659	11.1	0.007	<0.01
PA1777	oprF	1685	11.3	0.007	nt
PA3014	faoA	1313	8.9	0.007	nt
PA5565	gidA	269	1.8	0.007	nt
PA1801	clpP	494	3.4	0.007	17±8
PA1544	anr	736	5.3	0.007	0.05±0.03
PA2239	pslI	1256	9.0	0.007	nt
PA2775	-	2407	17.4	0.007	nt
PA5041	pilP	1730	12.6	0.007	nt
PA5490	cc4	1842	13.7	0.007	nt
PA3763	purL	1200	9.1	0.008	nt
PA2645	nuoJ	1579	12.3	0.008	nt
PA4223	-	223	1.9	0.008	nt
PA1802	clpX	117	1.0	0.008	nt
PA2853	oprI	1685	14.4	0.009	nt
PA1549	-	303	2.7	0.009	nt
PA4284	recB	1562	14.6	0.009	nt
PA0964	pmpR	2349	22.0	0.009	0.60±0.01
PA1005	-	3076	28.9	0.009	nt
PA1803	lon	391	3.7	0.009	nt
PA2397	pvdE	964	9.1	0.009	nt
PA4916	-	427	4.1	0.010	<0.01
PA4222	-	160	1.5	0.010	nt
PA5128	secB	721	7.0	0.010	nt
PA5001	-	902	8.9	0.010	nt
PA0596	-	710	7.4	0.010	nt
PA4697	-	2428	25.5	0.011	nt
PA4756	carB	1709	18.1	0.011	nt
PA2141	-	263	2.9	0.011	nt
PA3166	pheA	675	7.4	0.011	nt
PA5004	-	1126	12.4	0.011	nt

* all reads for each sample were normalized to 10,000,000 and divided by the gene length (Kb) and averaged between 2 input samples or 4 mouse infections.

** Selection ratio is the ratio of average normalized reads recovered from harvested wounds to average normalized input reads.

*** “nt” indicates not tested.

### Verification of Tn-seq results in mutant-by-mutant infection experiments

**Fig 4:**
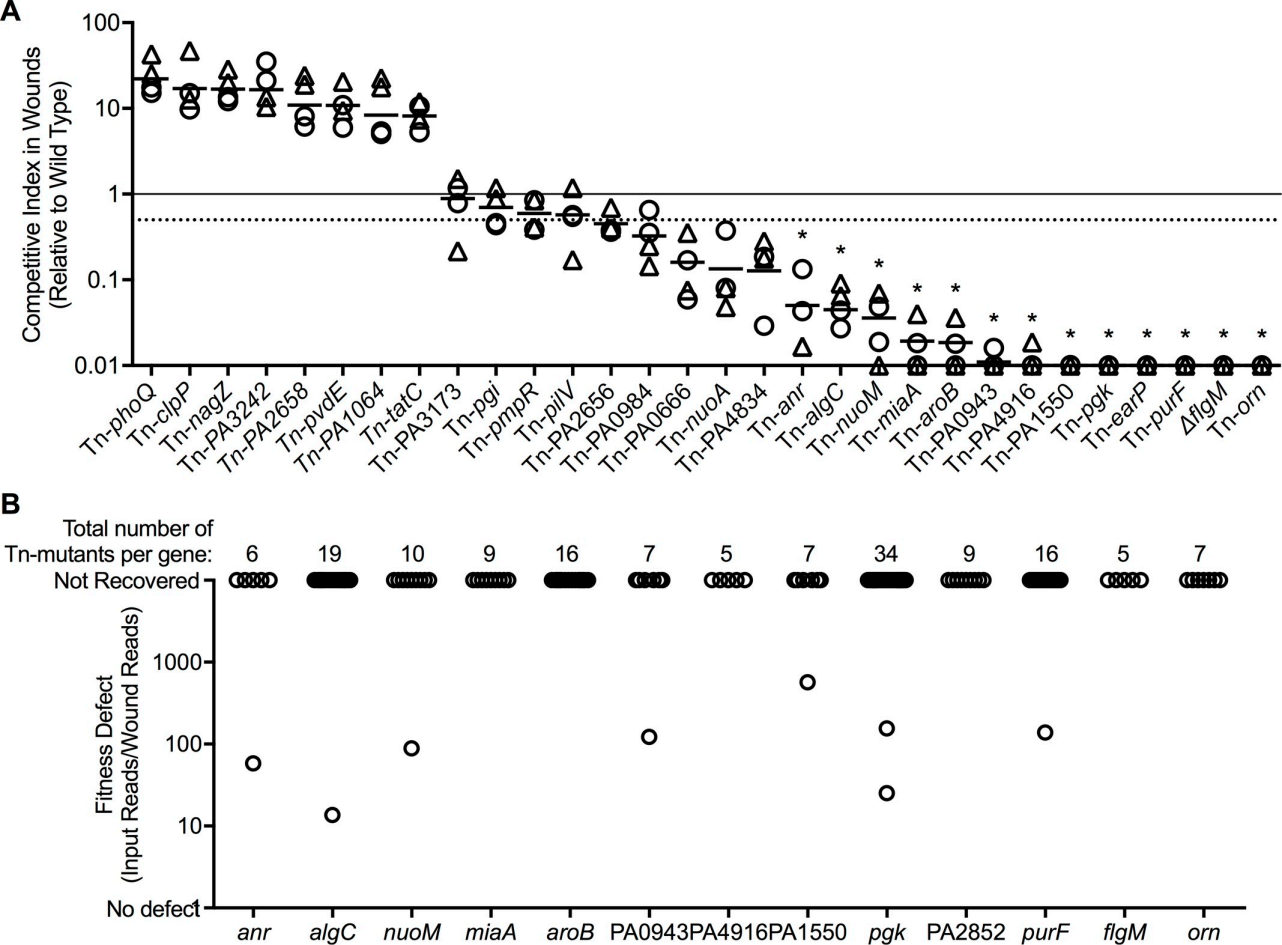
Confirmation of gene inactivations that compromise wound fitness. **A.** Mutants in 30 genes showing Tn-seq defects were tested in 1:1 competition with wild type, and mutant fitness in wound beds (triangles) or scabs (circle) was measured after five-days of infection. Dashed line indicates > two-fold defects in competition; * indicates competitive index significantly different than 1 (p<0.01) by one sample t-test. **B.** Fitness of individual transposon mutants in the 13 genes whose inactivation caused >10-fold impairments in wound fitness. Every gene verified to produce wound fitness defects was inactivated by at least five unique transposon mutants (points represent individual mutants). Almost every mutant in each gene exhibited similar fitness defects indicating that spontaneous secondary mutations were not likely responsible.

Tn-seq is a screening method that tests functions on a genomic scale, and errors can result from chance variations in the inoculum, mutant interference, or other problems. Thus, we tested independently-generated mutants from the PAO1 sequence-defined mutant library [30, 31] in 30 genes that showed strong defects in the Tn-Seq experiments (see Table 2 and Fig 4A). The majority of mutants with Tn-Seq defects (61%) that we tested also exhibited ≥ two-fold fitness defects in 1:1 competitions with wild-type PAO1 in mouse wounds (Fig 4A). Similar to observations in other studies [32–34] ~1/3 of the defects identified by Tn-seq were not reproduced in 1:1 competitions with wild-type PAO1.

Disruption of *flgM*, a negative regulator of flagellar synthesis genes [35], produced the largest fitness defect by Tn-seq, and was more than 100-fold less fit than wild type in 1:1 competition (Table 2 and Fig 4A). Inactivation of *flgM* results in overexpression of flagellin and lack of motility, however the *in vivo* defect could not be attributed to these two phenotypes (S5A Fig) because (1) the flagellated but non-motile mutant Δ*motABCD* was only 2-fold defective in wounds; (2) the non-motile Δ*fliC* did not exhibit a fitness defect; and (3) the Δ*flgM*Δ*fliC* mutant, which does not overexpress flagellin, was approximately 50-fold defective (S5A Fig). The finding that Δ*flgM*Δ*fliC* produced a similar fitness defect as Δ*flgM* argues against flagellin-induced inflammasome activation as a mechanism, as the Δ*flgM*Δ*fliC* does not produce flagellin. Instead it may be a consequence of previously undescribed phenotypes caused by *flgM* inactivation (S5 Fig and see below).

The mutant-by mutant verification experiments also confirmed the importance of other functions identified by Tn seq in wound fitness, including anaerobic growth (*anr*, *nuoA*, *nuoM*); metabolic functions (*aroB*, *pgk*, *purF*); and synthesis of cell envelope components (peptidoglycan recycling (PA0666), membrane proteins (PA0943 and PA4834), and LPS/extracellular polysaccharides (*algC*)) (Fig 4A). The fitness defect produced by inactivating anaerobic growth and metabolism genes is consistent with previous studies in chronic infection models [6, 25]. Seven of the 19 gene inactivations with verified wound defects have highly pleotropic effects (*orn*, *miaA*, *earP*, and PA2656), or unknown functions (PA1550, PA0943, PA4834, and PA4916) (Fig 4A).

We investigated several factors that could confound these results. First, we examined whether the transposon itself affected fitness by competing three transposon mutants with the corresponding unmarked deletions in the same genes. In one case (Tn-*phoQ*) the transposon mutant was more fit in murine wounds than its corresponding deletion, however in the other two cases (Tn-*fliC* and Tn-*pilV*) the transposon mutant exhibited comparable fitness to the unmarked strain. Thus, the transposon did not produce consistent fitness effects (S6 Fig).

Second, we investigated whether polar effects on neighboring genes or secondary mutations may have caused mutant fitness defects. However, as shown in S7 Fig, transposon insertions in downstream genes did not reproduce the identified defects. Furthermore, analysis of Tn-seq data indicated that many different transposon mutants in each gene exhibited similar defects (Fig 4B). These findings suggest that polar effects or secondary mutations were not likely responsible for the findings.

Third, we considered the possibility that other (non-*P*. *aeruginosa*) organisms that may be present in wounds could affect *P*. *aeruginosa* wound fitness. *Staphylococcus sp*. were present at >1% relative abundance of the total bacteria (but never more 15% relative abundance) in only 4 of 77 murine wound competition experiments (S8A Fig). In addition, we compared results of replicate experiments (testing the same *P*. *aeruginosa* mutant) in which *Staphylococcus* was and was not detected and found no differences in mutant recovery (S8B Fig). *Staphylococcus* was below the limit of detection (<1% of the population) in the other 75 of 77 murine wound competition experiments.

### Using mutants with high and low fitness to identify stressful conditions in wounds

The functional diversity of genes affecting wound fitness suggests bacteria may experience a range of disparate stress conditions during infection. Identifying these conditions is important as novel therapeutic approaches could augment *in vivo* stresses, or compromise the bacterial functions that counter them.

We explored the idea that *P*. *aeruginosa* mutants with differing wound fitness could be used to characterize the stress conditions operative *in vivo*. To this end, we exposed mutants verified to have high and low wound fitness (Fig 4A) to candidate conditions *ex vivo*, predicting that: (1) mutants with high wound fitness would function well in most candidate conditions relative to wild-type bacteria; and (2) a subset of mutants with low wound fitness would be compromised in each candidate condition, if the condition was representative of wounds.

**Fig 5:**
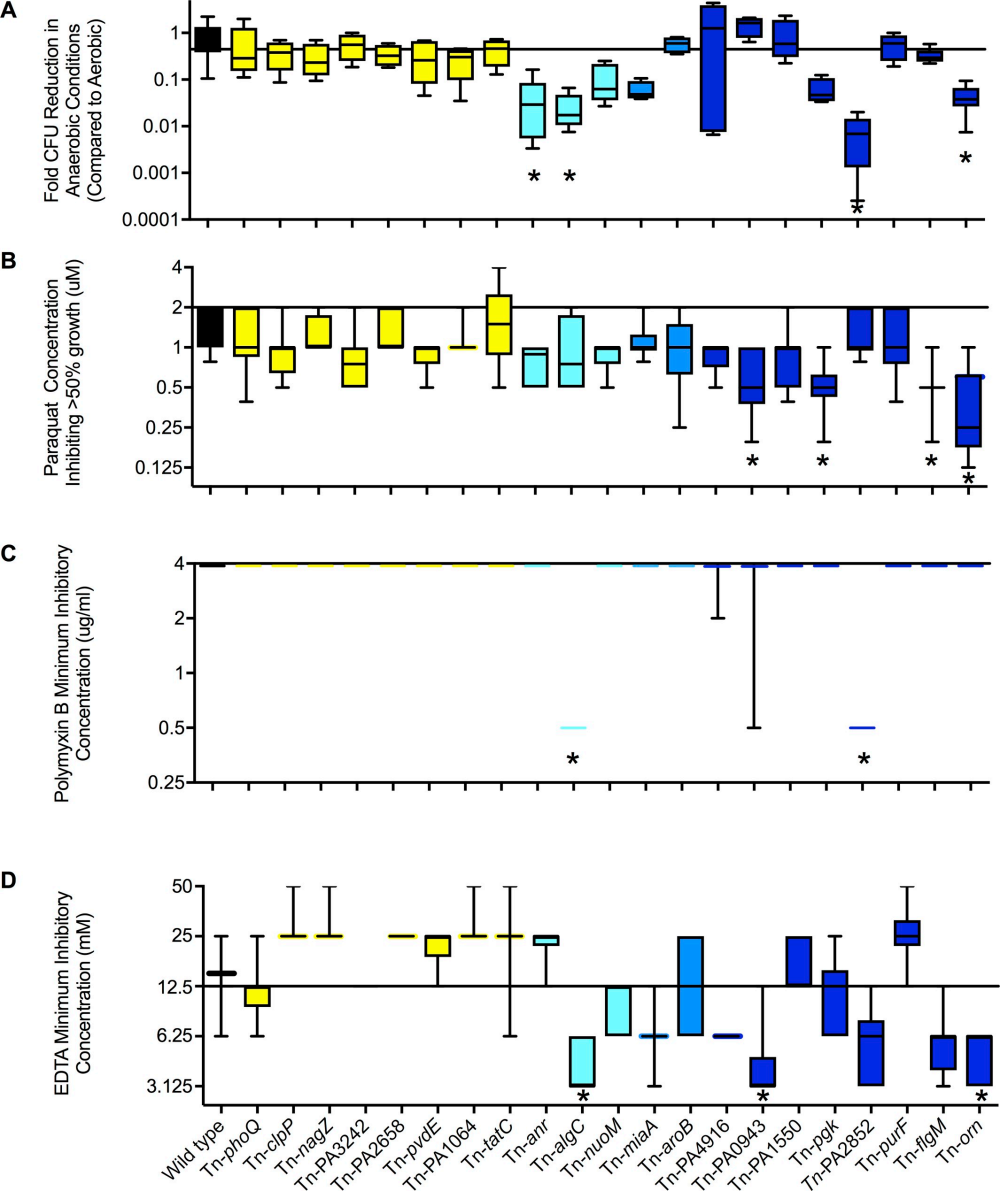
*In vitro* stress sensitivity of mutants with high and low wound fitness. Mutants with high (yellow bars) and low (blue bars, darker blue indicates more severe defects) wound fitness were tested for anaerobic growth capacity (**A**); and sensitivity to paraquat (**B**), polymixin B (**C**), and EDTA (**D**). Data is shown as box plots indicating the median, and 25^th^ and 75^th^ percentiles of at least three experiments (performed in duplicate with error bars showing the range. *indicates p<0.05 compared to wild type by non-parametric ANOVA (Kruskal-Wallis and Dunnet’s test).

To begin, we selected four stress conditions postulated to be operative in wounds: low oxygen availability, oxidative stress (represented by paraquat), and two membrane-stress conditions (represented by polymyxin and EDTA). Consistent with the predictions above, mutants with fitness greater than wild type in murine wounds did not exhibit significant defects in fitness (P>0.05 by Anova) in any of the 4 conditions (Fig 5, yellow boxplots). Also consistent, mutants with low wound fitness exhibited defects in some, but not all tested conditions (Fig 5, blue boxplots). For example, the mutant in PA2852 exhibited low fitness in the wound model, and after exposure to anaerobic conditions, EDTA, and polymixin B, but did not exhibit sensitivity to paraquat. Likewise, the mutant in PA0943 exhibited low fitness in the wound model, and sensitivity to EDTA and Paraquat, but did not exhibit significant defects in anaerobic growth or sensitivity to polymixin B.

These findings are consistent with the idea that different mutants may be sensitive to different stresses present within wounds. These data also suggest that anaerobic growth capacity, oxidative and membrane stress resistance may be determinants of bacterial fitness in wounds. We note that testing wound-defective mutants also revealed previously undescribed stress sensitivities in genes of known (*pgk* (Fig 5) and *flgM* (Fig 5 and S5C Fig)), and unknown (PA4916, PA1550, PA2852, PA0943) function (Fig 5).

### Fitness in high-density growth conditions correlates with wound fitness

The experiments above implicate anaerobic, oxidative, and membrane stress conditions as potentially important in wounds. The fact that these conditions can be produced by bacteria themselves [12, 36] led us to hypothesize that high-density bacterial growth might model the selective environment in wounds, in the absence of a host. We tested this hypothesis in three ways. For each test, we eliminated mutants with logarithmic-phase growth defects (S2 Table), as these mutants would likely be generally compromised.

**Fig 6:**
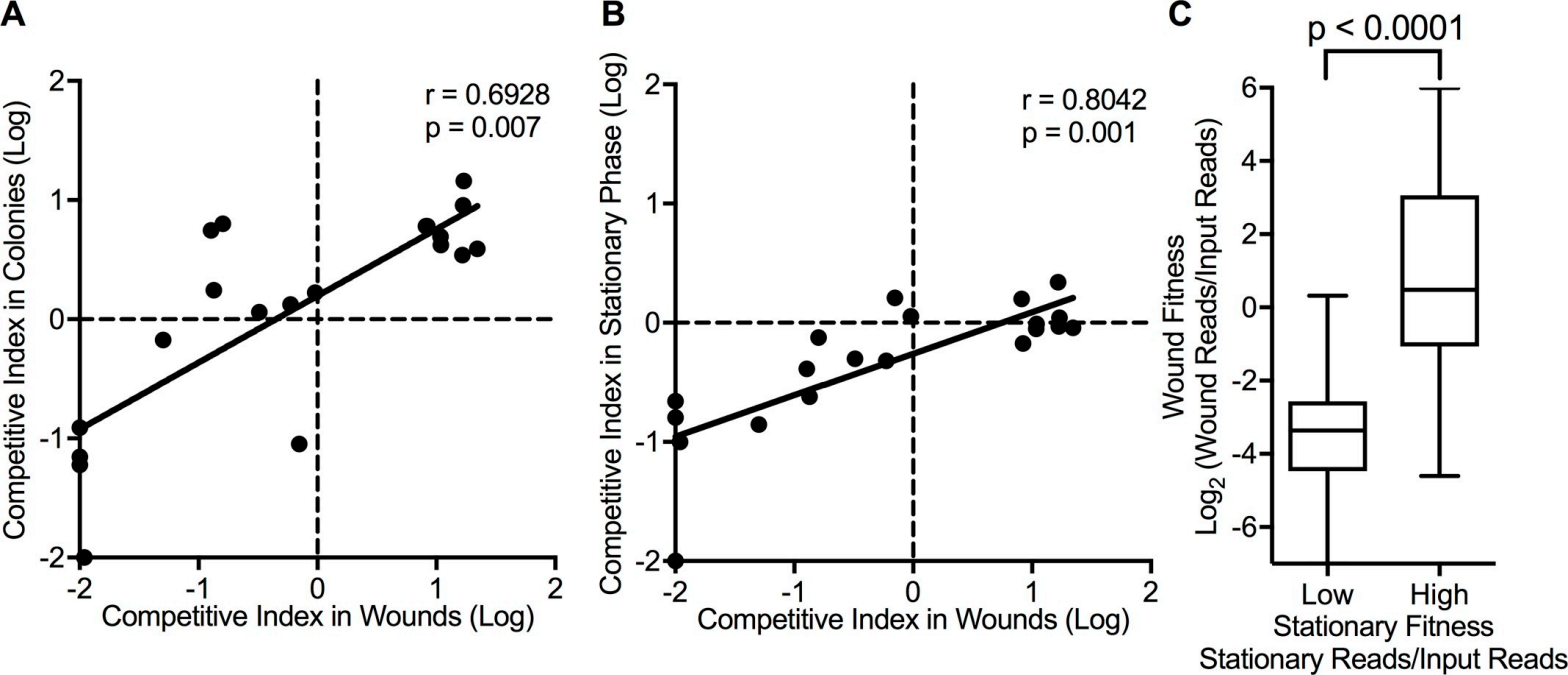
High-density growth and wound fitness correlate. **(A** and **B**) Relationship between the competitive index of mutants in colonies (**A**) or stationary phase cultures (**B**) and in wound infections. Spearman correlation and significance values were calculated using mean competitive index of each mutant. **C.** The wound competitive index of the 100 mutants exhibiting the lowest and highest stationary phase culture fitness (as measured by Tn-seq). Box plots indicate median, and 25^th^ and 75^th^ percentiles; error bars show the range of wound competitive index. Significance determined by Mann-Whitney analysis.

We first used a model in which bacteria grow at high density on filters placed on agar (“the colony model”). This model produces gradients of nutrients and wastes, and low oxygen availability, conditions characteristic of biofilms [25, 37]. However, biofilm formation functions such as adhesins, matrix production, and motility are dispensable in the colony model [37], as disaggregating forces of media flow or gravity are absent. As shown in Fig 6A and S2 Table, the wound fitness of mutants verified in mutant-by-mutant animal experiments was strongly correlated with their colony model fitness (p = 0.0007).

We also tested individual mutants with high and low wound fitness in stationary-phase liquid cultures, and found that stationary-phase and chronic-wound fitness correlated significantly (p<0.0001) (Fig 6B). Finally, we used Tn-seq to determine if the correlation between wound and stationary-phase fitness extended genome-wide. As shown in Fig 6C, the 100 mutants with the highest and lowest stationary phase fitness exhibited analogous fitness in chronic wounds (p<0.0001). Thus, the genetic determinants of bacterial fitness in wounds are similar to those needed in high-density *in vitro* growth conditions that do not involve host interactions or biofilm formation.

## Discussion

This study advances understanding of the *P*. *aeruginosa* functions needed to initiate and maintain chronic wound infections and the stress conditions that affect bacterial fitness in wounds. A key finding was that many virulence functions were inactivated in human wound infection isolates, and only one of the virulence functions we tested affected bacterial fitness in murine chronic wounds. Type three secretion was the exception as our mutant by mutant wound experiments found that inactivation of type three secretion compromised bacterial fitness in wounds, consistent with previous work [6]. Our finding that many *P*. *aeruginosa* virulence factors could be inactivated without reducing bacterial fitness in wounds, along with consistent findings from other studies [3, 6–8], suggest that chronic wounds sites may be relatively permissive environments in which bacterial nutrient acquisition and growth may not depend upon the invasive or toxic functions. A permissive host environment could also explain why low virulence “commensal” organisms commonly inhabit wounds [38, 39]. It is notable that isolates from chronic CF infections have been shown to exhibit defects in many of the same virulence functions.

However, our experiments do not indicate that wound environments are stress-free. We found that genes mediating resistance to anaerobic and membrane stress, and certain metabolic pathways were critical in infection. Independent experiments investigating the phenotypes of mutants with high and low wound fitness corroborated the importance of anaerobic, oxidative, and membrane stress in wounds. Previous work using Tn-seq and gene expression analysis in wounds also found nutritional functions to be important (including those mediating purine and amino acid biosynthesis) [6].

Our experiments using colonies and stationary phase cultures suggested that the selective environment in wounds could be approximated by high-density growth conditions in the absence of a host. This finding is consistent with work showing that the *P*. *aeruginosa* gene expression profile in murine wounds resembles that in high-cell density colonies [25]. Taken together, these findings raise the possibility that the predominant stresses on organisms in wounds may be applied by the bacteria themselves, rather than the host. It is also possible that overlapping functions counter bacterial and host-applied stresses.

Our data also sheds light on the role of biofilms in chronic infections. Our finding that isolates that successfully infect human wounds are often biofilm-deficient *in vitro* is similar to results using CF isolates [14]. We also found that genetic manipulations that markedly changed the capacity of *P*. *aeruginosa* to form biofilms did not produce corresponding effects in murine wounds. These results argue against the importance of biofilm formation functions, that are defined using laboratory systems, in these chronic infections. This is consistent with a previous report that expression of the predominant exopolysaccharide in PAO1, psl, is not increased in the chronic murine wound environment [6] despite the appearance of the bacteria in multi-cellular aggregates [9].

Biofilm formation functions may be dispensable in CF and wounds because the non-invasive and resistant bacterial phenotypes characteristic of chronic infection are a consequence of host conditions [40]. For example, cell aggregates may be a default growth mode in thick polymer-rich exudates present in CF airway and wounds [17, 18]. It is also possible that active mechanisms of aggregation are needed *in vivo*, but these functions do not overlap with biofilm formation functions identified *in vitro*. Nevertheless, the correlation between the fitness of mutants in wounds and high-density growth conditions suggest that the physiological stresses inherent to biofilm growth may be relevant in wounds, even if biofilm formation functions are not.

Our study had several limitations. First, the clinical isolates we studied from acute infections all came from different patients, so we were unable to determine if isolate populations from individual subjects exhibited diversity in biofilm formation or virulence factor expression as wound isolates did. Second, limited clinical information was available about subjects from whom wound isolates were collected. Third, while the Tn-seq experiments comprehensively examined the effect of most non-essential genes, we did not test all identified virulence factors in mutant-by-mutant wound infection experiments. Thus, it is possible that some virulence functions (in addition to type three secretion) affect bacterial fitness in murine wounds.

Fourth, the *in vivo* Tn-seq screening and verification experiments involved competitions between strains to identify fitness defects. This is a limitation, as some defects can be compensated for by the wild type in competitions. However, we found that all of the isolates from two wounds were protease-negative and all of the isolates from three wounds were rhamnolipid negative. Thus the subpopulations expressing these functions are very small, if they exist. Furthermore, the strain lacking both quorum sensing systems, and thus many secreted virulence factors, exhibited no significant defect in murine wounds in pure culture. These findings argue against the interpretation that subpopulations of bacteria producing virulence factors generally compensated for defective mutants in our experiments.

Fifth, we note that while the biofilm and virulence gene mutants we studied were engineered deletions, we also used transposon mutants in some experiments to verify the Tn-seq results. Thus, it is possible that in some cases the transposon, or its insertion location, may have affected results. However, control experiments showed that the transposon did not generally affect *in vivo* fitness. Furthermore, the fitness defects of genes verified to be important for infection were replicated by many different mutants in each gene. These included the many independent mutants (in each gene) generated in the Tn-seq libraries (see Fig 4B and S7 Fig) and the independent sequence-defined transposon mutants [30, 31] used in verification experiments.

Sixth, we used a well-defined laboratory strain, PAO1, which likely differs both in genomic content and gene expression from clinical wound isolates. However, despite these differences the virulence and biofilm phenotypes of many clinical wound isolates were generally corroborated by our experiments with PAO1 in the murine wound model.

Finally, we note that the murine infection model does not represent all relevant conditions of human wounds. In particular, our murine infection model was used primarily to examine the necessity of genes early in chronic infection (within the first five days) which is shorter than the six weeks of infection which can be sustained by this model or the months to years of infection observed in human wounds.

Our study also has had strengths that increased confidence in the results. We verified the fitness defects identified by Tn-seq with individual mutants. Such verifications are critical, as we found that ~1/3 of the defects identified by Tn-seq were not reproducible in mutant-by-mutant assays. This is consistant with other studies which have shown similar levels of reproducibility from Tn-seq screens [32–34]. In addition, results with clinical isolates and experimental murine infections gave generally concordant results regarding the importance of virulence and biofilm formation functions in wounds. Likewise, tests with wound-defective mutants and independent Tn-seq experiments both suggested that bacterial fitness in high-density growth conditions and wounds were strongly correlated. Finally, the wound model had key strengths. Diabetic mice were used, and diabetes is a key factor in human wound pathogenesis [41]. Furthermore, *P*. *aeruginosa* inhibited wound-healing in the model, which directly links infection to disease.

In addition to increasing understanding of wound infection pathogenesis, this study highlights challenges and opportunities in therapeutics development. One novel approach seeks to target infection pathogenesis functions, rather than trying to kill or inhibit bacteria [42]. However, our data suggest that targeting biofilm formation and many virulence functions may not be useful in chronic infections. Our finding that the functions needed in wounds and high-density cultures overlap suggests that separating basic growth and chronic infection pathogenesis functions may be difficult.

Our findings also point toward opportunities. The fact that stationary phase cultures and colonies generally represented the selective environment of wounds raises the possibility that these systems could be used as an initial screen for potential therapeutics, or therapeutic targets. Such approaches could offer “first-pass” efficiency and throughput advantages as compared to animal or cell-based models. Finally, our work suggests parallels in the pathogenic mechanisms of two of the most significant chronic bacterial infections: those afflicting people with chronic wounds and cystic fibrosis. This is important because animal models that faithfully represent most chronic bacterial infections are lacking, and progress may be accelerated if therapeutic targets relevant for multiple chronic infections could be identified.

## Materials and methods

### Ethics statement

Debridement samples were collected from adults at the Southwest Regional Wound Care Center (Lubbock, Texas) in accordance with Western Institutional Review Board protocol number 20062347. Patients provided written informed consent prior to collection of samples. All animal experiments were conducted with University of Washington Animal Care and Use Committee approval in compliance with the National Institutes of Health guide for the Care and Use of Laboratory Animals under IACUC Protocol Number 4113–01.

### Growth conditions and strains

Strains used are listed in S4 Table. Cultures were grown on TYE agar (10 g peptone, 5 g yeast extract, 8 g NaCl per liter) or in LB broth or agar (10 g peptone, 5 g yeast extract, 10 g NaCl per liter) at 37°C. Strains were stored in LB + 20% glycerol at -80°C. Gene deletions were created by homologous recombination as described previously [43]. Transposon mutants were obtained from the *Pseudomonas aeruginosa* mPAO1 transposon mutant library [30, 31] and the location of each transposon was verified by PCR following the guidelines from the library website (http://www.gs.washington.edu/labs/manoil/libraryindex.htm). Clinical isolates were confirmed to be *P*. *aeruginosa* by growth and morphology on Pseudomonas Isolation and Cetrimide Agar, as well as with *P*. *aeruginosa* specific PCR [44]. Previously identified environmental and acute isolates were obtained from the Hauser and Salipante Lab collections [45–47].

### Clinical wound, environmental, and acute isolate analysis

Patients undergoing treatment for chronic wound infections were selected for this study based on previous detection of *P*. *aeruginosa* in wounds by culture and/or PCR for months to years prior to collection. Wound debridement samples were obtained at the Southwest Regional Wound Care Center during the course of standard clinical wound care, and were transported by overnight mail at 4°C. Samples were from patients with varying wound etiologies including, but not limited to diabetic ulcers, pressure sores, circulatory disorders, and trauma.

Samples were homogenized in 1 ml of PBS at 26,000 rpm for 15 seconds (Polytron PT3100 Benchtop Large-Scale Homogenizer, Capitol Scientific Inc., Austin, TX) prior to plating on MacConkey agar (BD Difco) and Pseudomonas Isolation agar (Remel). Twenty-four to forty-eight hours after plating, up to 96 presumptive *P*. *aeruginosa* isolates per sample were picked, grown in LB overnight, and stored at -80^0^ C in LB + 20% glycerol. Up to five debridement samples taken from a single debriedment session were plated for each wound resulting in up to 480 isolates per wound. Isolates were confirmed to be *P*. *aeruginosa* by growth and morphology on Pseudomonas Isolation Agar (Remel) and Cetrimide Agar (Hardy Diagnostics) as well as *P*. *aeruginosa* specific PCR as described in [48].

Protease activity was determined by detecting a clear halo surrounding growth on skim milk agar plates (4% milk) [49]. Rhamnolipid production was determined by detecting a white halo on Rhamnolipid agar plates after 24 hours growth at 37°C and four days at room temperature in the dark as previously described [50]. Swimming motility was identified by bacterial spreading from a stab inoculation point sufficient to cover the surface of 0.3% LB agar in a 96 well plate after 24 hours at 30°C. Twitching motility was determined by bacterial spreading along the plastic surface of petri plates > 0.5mm beyond the inoculation point for all positive samples from a stab inoculation point on 0.5% LB agar. Swimming motile (wild type PAO1) and non-motile (PAO1 Δ*fliC*) as well as twitching motile (wild type PAO1) and non-twitching (PAO1 Δ*pilA*) strains were tested repeatedly under these conditions across multiple experiments and batches of media. In all cases the positive controls tested positive and the negative controls tested negative. All assays were performed at least twice to confirm isolate phenotype.

*In vitro* biofilm formation was assayed as previously described [21]. Two hundred μl of a logarithmic culture was diluted to an OD_600_ of 0.05 and incubated in LB broth without shaking in 96 well microtiter plates for 22 hours at 37°C covered with Breath Easy membrane (USA Scientific). After incubation, total biomass (planktonic and biofilm) was measured by OD_600_. Planktonic cells were removed and the biofilm attached to the plastic was stained with 0.1% crystal violet. Crystal violet was eluted with 95% ethanol and measured by OD_595_. The ability to form biofilms was determined by the ratio of OD_595_ (biofilm) to OD_600_ (total biomass) to account for differences in bacterial growth. Biofilm-deficient strains were defined as having a ratio of OD_595_/OD_600_ less than 50% compared to wild-type mPAO1.

Protease secretion, rhamnolipid production, twitching motility, swimming motility, and biofilm formation from acute, environmental, and chronic wound sources were compared using Fisher exact test. In order to allow for comparison between the chronic wound samples, for which we had 100s of isolates per wound, and the acute and environmental samples, the data from the chronic wounds was first condensed to either positive or negative based on the frequency of positive isolates. If >50% of isolates from a single wound were positive for a phenotype then the wound was given a negative score. If <50% of isolates for a single wound were positive for a phenotype then the wound was given a positive score. The frequency of positive wounds was then compared to the frequency of positive environmental or acute isolates for each phenotype using Fisher exact test.

### Murine wound infections

A 6-mm full-thickness excisional wound was created on the dorsal skin of 10–14 week old diabetic female mice (db/db; BKS.Cg-Dock7^m^ +/+ Lepr^db^/J) (Jackson Laboratory, Bar Harbor, ME) as previously described [9, 22]. Mice were inoculated with planktonic *P*. *aeruginosa* two days after wounding. Wounds were covered with tegaderm (3M) for two days post infection. Unless otherwise noted, mice were euthanized five days post infection and the scab and wound were excised as previously described [9, 22].

### Histology and wound healing measurements

Wound areas were documented using Nikon D1 digital camera equipped with a Micro Nikkor macrolens and dual electronic flash (Nikon, Tokyo, Japan). Wounds including 0.25 cm margin of surrounding skin were removed and bisected. Six micron paraffin sections were stained with hematoxylin & eosin (H&E), and evaluated for re-epithelialization. The original wound edge was determined as the point in which subcutaneous fat tissue, mature collagen, or mature hair follicles were absent. Percentage of wound closure was calculated using the following equation: 100%—(distance between re-epithelized tissue/distance between original wound edge)*100. Harvested scabs and wounds were homogenized in 1 ml of PBS at 26,000 rpm for 15 seconds (Polytron PT3100 Benchtop Large-Scale Homogenizer, Capitol Scientific Inc., Austin, TX) prior to dilution in PBS and plating on LB agar. *Pseudomonas* colonies were confirmed by growth on PIA and colony morphology. Only *P*. *aeruginosa* isolates were used to calculate the ratio of mutants to wild type. Any non-*Pseudomonas* isolates were grown on Manitol Salts Agar and confirmed to be *Staphylococcus spp*. Significant changes in healing or CFU were identified by Mann-Whittney.

### Tn-seq

mPAO1 was mutagenized with transposon T8 (ISlacZ*hah-*tc) by conjugation with donor *E*. *coli* to create a mutant pool as previously described [30]. Mutant pools were used as the inoculum for wound infections, the colony model, and stationary phase fitness experiments. To recover bacteria after wound infections, the wound beds and scabs were excised five and seven days after infection, homogenized, and genomic DNA isolated (Qiagen DNEasy Blood and Tissue Kit). Samples were prepared for Tn-seq using either the circle method (for the Tn-seq on chronic mouse wound) [32] or the TdT method (for the Tn-seq to compare mouse wound and stationary phase) [51].

For the Tn-seq circle method A-Tailing (NEBNExt dA-Tailing Kit with Klenow Fragment), adaptor ligation (NEB Quick ligase), and DNA cleanup (AMPure XP Beads) were performed in a single tube. Size selection, circularization, exonuclease digestion, and junction PCR were performed as previously described [32]. Sequencing of samples was performed using Illumina GAIIx.

To prepare samples using the TdT method, a poly C-tail was added to end repaired DNA using terminal transferase (Promega), dCTP, and ddCTP (Affymetrix) prior to purification using Perfoma DTR gel filtration columns (Edge Bioxystems). MiSeq adaptor sequences and index sequences were added using two rounds of PCR with KAPA HiFi (Kapa Biosciences) as previously described [51]. Sequencing was performed on MiSeq using the 50-cycle kit (Illumnia).

### Tn-seq computational analysis

Sequences were analyzed using the custom Perl scripts described previously [32]. Analyzed reads from each sample were normalized to 10,000,000 reads per sample, and transposon insertions that mapped to either the beginning 5% or the last 10% of their open reading frames were excluded. The combined number of reads per mutant was normalized for gene length. Any genes that were not highly represented in the input pool (less than two hits per gene in both of the input pool samples or 100 reads per KB) were eliminated from further analysis. The wound fitness of the remaining ~5000 genes was calculated as the ratio of the average number of reads (normalized to gene length) from the wound samples, compared to the average number of reads from the input pool. A histogram of the log2-transformed fitness ratio was plotted and genes significantly defective in fitness (p<0.01) were identified.

To compare Tn-seq results between the mouse wound and stationary phase, the wound Tn-seq experiments were repeated so that the same mutant library could be used. We eliminated mutants with impaired exponential phase growth (as determined by Tn-Seq) from analysis as these mutants would likely show general defects. To allow for comparison between experiments, the ratio of reads in the output compared to the input was normalized between experiments. In order to test for correlation in fitness in stationary phase and the mouse wound we focused on the 100 genes with the lowest and highest ratio of reads in stationary phase to reads in the input. The average fitness in the mouse was then calculated for the 100 genes with low stationary fitness and the 100 genes with high stationary fitness and compared using mann-whitney analysis.

### *In vitro* and *in vivo* competition experiments

For *in vivo* competitions, overnight cultures on TYE agar were resuspended in PBS and washed twice prior to resuspension in PBS at OD_600_ = 1. Ten μl of the inoculum (~1X10^7^ CFU) was pipetted onto wounds two days after wounding and the remaining culture was serially diluted and plated on LB for determination of CFU and the ratio of mutant to wild-type *P*. *aeruginosa*. The scabs were removed from the wounds five days after infection and the scabs and wounds were homogenized individually in 1 ml PBS, and serial dilutions were plated for bacterial enumeration. For each sample the ratio of mutant to wild-type *P*. *aeruginosa* was determined for 100 colonies by noting the bacterial phenotype (for unmarked motility mutants) or tetracycline resistance (for transposon mutants). Isolates from the mice were confirmed to be *P*. *aeruginosa* by growth on PIA. Competitive index was calculated by dividing the ratio of mutant to wild type in the mouse samples to the ratio of mutant to wild type in the input. Significance was determined using one sample t-test with a cutoff of p<0.05. For the *wspF* and *wspF*/*pel*/*psl* mutants the scab and wound were homogenized together and then frozen. Total DNA was isolated (DNeasy, Qiagen) and tested by qPCR to measure relative abundance of strains with intact *wspF* (indicating wild-type strain) to *wspF* using primers that quantified total *P*. *aeruginosa* DNA and full-length *wspF*.

For *in vitro* competitions to analyze fitness in logarithmic, stationary phase, and the “colony model”, cultures were prepared as above except that wild-type mPAO1 was marked with CTX::lacZ. Logarithmic and stationary phase studies were performed on cultures in 3 ml LB grown shaking at 37°C for four or 22 hours respectively. For the colony model [37] samples were inoculated onto a 13mm 0.22 micron polycarbonate filter (GE) and allowed to grow for 48 hours on LB agar. The competitive index was calculated as above by comparing the number of white (mutant) colonies to blue (wild type + CTX::lacZ) colonies when grown on LB + 25 ug/ml X-gal. Significance was determined using students t-test with a cutoff of p<0.05.

### Isolate stress sensitivity tests

Anaerobic growth capacity was determined by comparing the CFU counts of cultures grown in LB broth supplemented with 100 mM KNO_3_ grown anaerobically (BD Gas Pak EZ system) or aerobically (with shaking) for 22 hours. Paraquat (methyl viologen) and EDTA sensitivities were measured by diluting mid-log cultures to an OD_600_ of 0.005 in wells containing two-fold dilutions of paraquat or EDTA. The inhibitory dose 50% (ID50) is the average lowest concentration of paraquat at which the OD_600_ was less than half of the OD_600_ of the no-paraquat control wells after 20 hours of growth. The MIC in EDTA-containing media is the average lowest concentration of EDTA at which growth was inhibited. The polymyxin B MIC was determined by spotting 10 μl of 5X10^6^ CFU of each tested isolate (based on OD_600_) on plates containing two-fold dilutions of Polymyxin B and measuring growth after 16 hours at 37°C. Significant changes in sensitivity were determined using students t-test with a cutoff of p<0.05.

## Supporting information

S1 Fig Example virulence phenotypes of clinical isolates. A) Protease-positive isolates exhibited clearing around colonies on skim milk agar plates, whereas no clearing was detected around protease-negative isolates. B) Rhamnolipid production was detected by the presence of a white halo surrounding the colonies when grown on rhamnolipid-indicating media. Isolates unable to produce rhamnolipids appeared blue on this media. C) Twitching motility was detected by noting bacterial spreading along the bottom of a petri dish containing LB + 1% agar. To aid in visualization, the agar was removed and the bacterial biomass was stained with crystal violet. D) Swimming motility was detected by the presence of bacterial growth extending from the inoculation point on LB + 0.3% agar.
(PDF)

S2 Fig The motility and biofilm formation phenotypes of isolates were often discordant. Isolates from human wounds were assayed for ability to form biofilms in microtiter wells, swimming motility, and twitching motility. A) Although the majority of the biofilm positive isolates were also positive for swimming, 13 isolates from wound two were swimming negative but still formed biofilms. B) Despite most of the chronic wound isolates lacking twitching motility, many of these isolates formed biofilms.
(PDF)

S3 Fig No significant weight change was observed in mice with wound infections. Weight (in grams) was measured for challenged (black line) and unchallenged (blue line) mice at the time of wounding (2 days before infection), the time of infection, and five and 11 days after infection. Data are means and standard deviations; eight mice were infected in each group. No significant difference in weight was observed at any time point.
(PDF)

S4 Fig The quorum sensing mutant establishes wound infections and bacterial yields are comparable to wild-type *P. aeruginosa*. After five days of infection there was no significant difference (by student’s t-test) in the CFUs of *P*. *aeruginosa* recovered from wounds infected with wild-type and quorum sensing mutant (Δ*lasR*Δ*rhlR*) *P*. *aeruginosa*. Bar indicates geometric mean of CFU counts.
(PDF)

S5 Fig The *flgM* gene, but not swimming motility is required for fitness in wounds and in *in vitro* stress conditions. We conducted studies to investigate mechanisms explaining the severe wound fitness defect of the *flgM* mutant. Inactivation of *flgM* increases expression of flagellin (encoded by *fliC*), and Δ*flgM P*. *aeruginosa* produce an abnormal flagella that is non-motile.
A) The wound fitness defect of Δ*flgM* could not be explained by its swimming motility defect, as the non-motile Δ*fliC* mutant exhibited no fitness defect in wounds. Likewise, the wound fitness defect of Δ*flgM* could not be explained by the presence of a non-motile flagella, as Δ*motABCD P*. *aeruginosa* (which also has a non-motile flagella) exhibited near wild-type levels of wound fitness. We also tested a Δ*fliC/*Δ*flgM* double mutant, which lacks both flagella and FlgM’s regulatory actions, and found that the Δ*fliC/*Δ*flgM* mutant had a wound defect comparable to that of the Δ*flgM* strain. These experiments implicate some effect of FlgM (other than on motility or *fliC* regulation) in Δ*flgM’s* wound fitness defect. * indicates competitive index significantly different than 1 (p<0.05) by one sample t test.
B) We tested flagellar mutants for oxidative stress sensitivity. The Δ*flgM* and Δ*fliC/*Δ*flgM* mutants exhibited increased paraquat sensitivity as measured by the minimum concentration of paraquat required to inhibit 50% of growth (IC50). The other non-motile mutants tested (Δ*fliC* and Δ*motABCD*) were less sensitive to paraquat than Δ*flgM* and Δ*fliC/*Δ*flgM*. The x-axis is set to cross the y-axis at the value of wild-type *P*. *aeruginosa*’s paraquat IC50 for ease of visualization.
C) We used Biolog Phenotypic microarrays (PMs) to identify additional stress sensitivities caused by *flgM* inactivation. Graphs show respiratory activity (Y-axis) as a function of time (X-axis) of wild type *P*. *aeruginosa* and the Δ*flgM* strain. Respiratory activity is a surrogate for growth in the indicated stress conditions. Respiratory activity of wild-type *P*. *aeruginosa* is shown by red-shaded areas; the Δ*flgM* strain is shown by green-shaded areas; and areas where red and green overlap appear yellow. Graphs in which the red shading exceeds the green shading indicate stress conditions in which the Δ*flgM* mutant is impaired relative to wild type (see www.biolog.com/pdf/pm_lit/PM1-PM10.pdf and www.biolog.com/pdf/pm_lit/PM11-PM20.pdf). For some conditions Biolog does not supply the actual concentration of the agent used. For these conditions, graphs representing low, medium and high concentrations are indicated.
(PDF)

S6 Fig The presence of the transposon does not confer a uniform fitness effect. Transposon mutants were competed against unmarked deletions in the same gene. The competitive index of the transposon mutant relative to the unmarked deletion in the same gene in scabs (circle) or wound beds (triangle) five days after infection indicates no consistent trend of increased (CI>1) or decreased (CI<1) fitness due to the presence of the transposon amongst the three mutants tested. * indicates that there is a significant difference in fitness between the unmarked deletion and the transposon mutant by the 1-sample t-test.
(PDF)

S7 Fig Secondary mutations and polar effects likely had minimal effects on mutants’ wound fitness. We used Tn-seq data to determine if polar effects on neighboring genes could explain the defects of mutants verified to have greater than ten-fold impairments in wound fitness (see verification experiments in Fig 4). Verified wound-defective mutants are indicated by yellow highlighting, and genes immediately up- and down-stream are not highlighted. Because the transposon we used in Tn-seq experiments is more likely to cause polar effects on downstream genes when inserted in the reverse orientation [53], we marked forward- and reverse-oriented transposon insertions with black and blue points respectively. Of the 11 verified wound-defective mutants analyzed, the fitness defects in seven (*algC*, *anr*, *miaA*, *purF*, PA0943, PA4916, and *pgk)* were not likely due to polar effects (see panels A-C). We cannot rule out polar effects for four verified wound-defective mutants (*nuoM*, aroB, PA1550, and *earP*) as the transposon mutant in the downstream gene was also compromised for wound fitness (see panel D). A)The transposon insertions in the genes downstream of *algC*, and *anr* (*argB* and *apt*) produced less severe wound fitness defects than the insertions in *algC* and *anr*. B) The gene downstream of *miaA* had no inserts. C) There was no downstream gene in the operons of *purF*, PA0943, PA4916, and *pgk*. D) The transposon mutant in the genes downstream of *nuoM*, aroB, PA1550, and *earP* were also compromised for wound fitness. However, for three of these four genes the downstream gene (*nuoN*, *aroK*, and *efp*) is in the same pathway as the verified wound-defective mutant (*nuoM*, *aroB*, and *earP*). Thus, the verified wound-defective mutant, and the transposon in the downstream gene may independently affect wound fitness.
(PDF)

S8 Fig The presence of Staphylococcus does not confer a uniform fitness effect. A) In the 1:1 competitions between wild-type and mutant *Pseudomonas*, only four wounds were contaminated with *Staphylococcus*. B) Competitive index information the three *P*. *aeruginosa* mutants for which one replicate experiment was contaminated with <1% *Staphylococcus*, and the other replicate experiment was not. The competitive index of the transposon in scabs (circle) or wound beds (triangles) for each replicate is shown. The x-axis indicates if that wound was contaminated with < 1% *Staphylococcus* (based on growth on Mannitol Salts Agar).
(PDF)

S1 Table Tn-seq data from chronic mouse wound indicating the number of hits (number of unique transposon sites) and reads (number of times a transposon in this gene was detected). (XLSX)

S2 Table Competitive index of mutants in mouse wound, logarithmic culture, colony, and stationary phase. (PDF)

S3 Table Tn-seq data indicating number of reads per gene from the input pool, 5-day chronic murine wound infection, stationary phase (22 hour), and logarithmic phase (4 hour). Data for each replicate is shown.
(XLSX)

S4 Table Strains used in this study. (PDF)
