# Supplementary figures and images for: Bacterial fitness in chronic wounds appears to be mediated by the capacity for high-density growth, not virulence or biofilm functions
Source: PLoS Pathog. 2019 Mar 20;15(3):e1007511. doi: 10.1371/journal.ppat.1007511 (PMC6448920; doi:10.1371/journal.ppat.1007511)

**S1 Fig.**

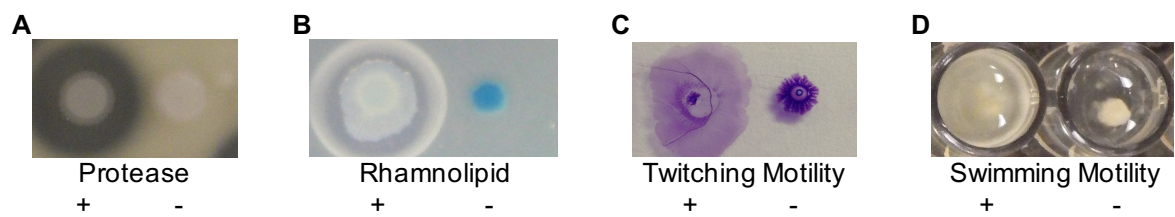

Supplement: S1 Fig — A) Protease-positive isolates exhibited clearing around colonies on skim milk agar plates, whereas no clearing was detected around protease-negative isolates. B) Rhamnolipid production was detected by the presence of a white halo surrounding the colonies when grown on rhamnolipid-indicating media. Isolates unable to produce rhamnolipids appeared blue on this media. C) Twitching motility was detected by noting bacterial spreading along the bottom of a petri dish containing LB + 1% agar. To aid in visualization, the agar was removed and the bacterial biomass was stained with crystal violet. D) Swimming motility was detected by the presence of bacterial growth extending from the inoculation point on LB + 0.3% agar. (PDF) [file ppat.1007511.s001.pdf]

S2 Fig.

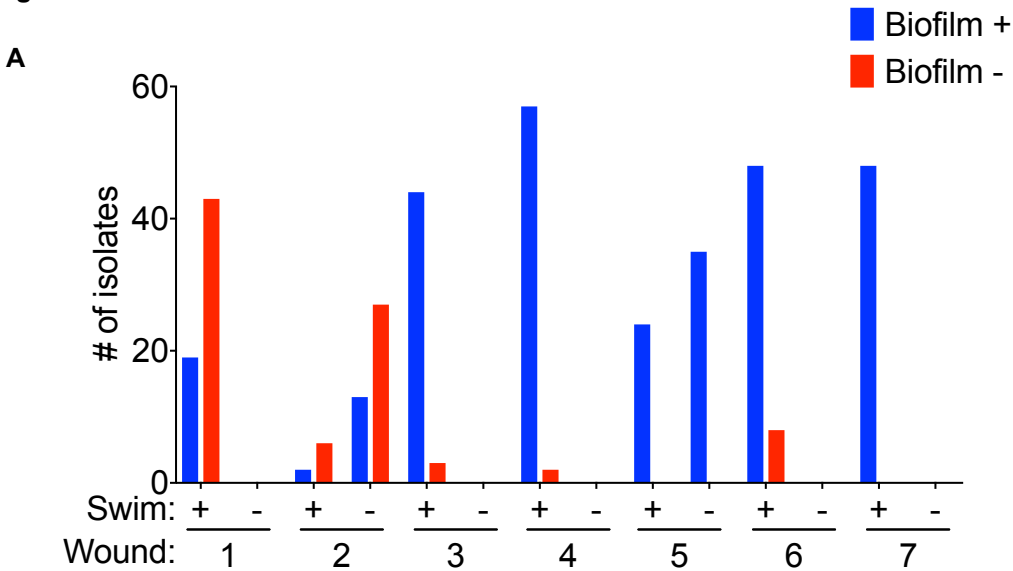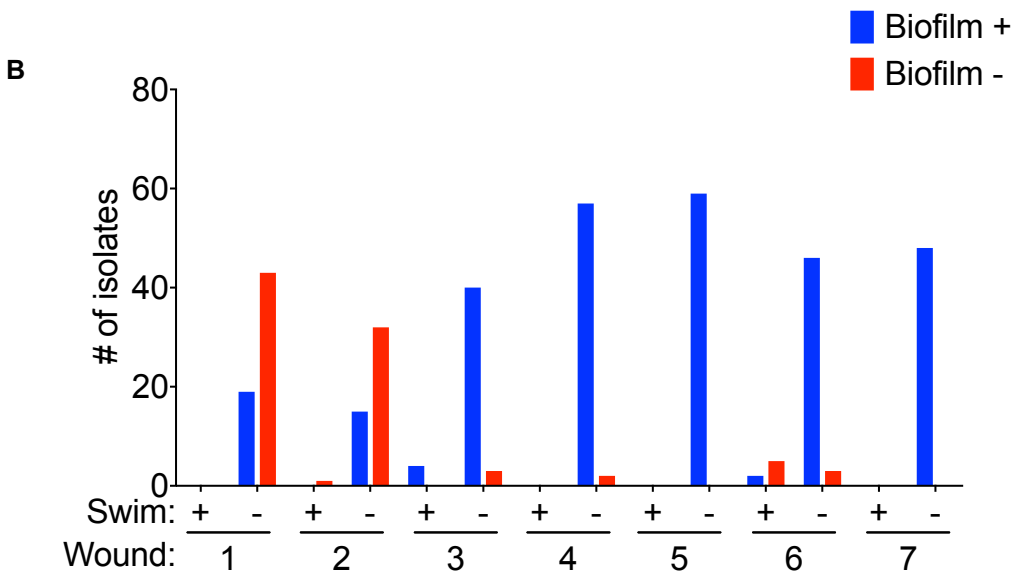

Supplement: S2 Fig — Isolates from human wounds were assayed for ability to form biofilms in microtiter wells, swimming motility, and twitching motility. A) Although the majority of the biofilm positive isolates were also positive for swimming, 13 isolates from wound two were swimming negative but still formed biofilms. B) Despite most of the chronic wound isolates lacking twitching motility, many of these isolates formed biofilms. (PDF) [file ppat.1007511.s002.pdf]

S3 Fig.

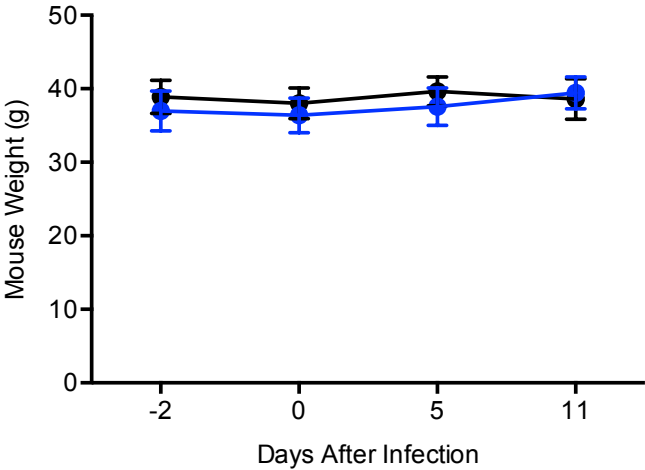

Supplement: S3 Fig — Weight (in grams) was measured for challenged (black line) and unchallenged (blue line) mice at the time of wounding (2 days before infection), the time of infection, and five and 11 days after infection. Data are means and standard deviations; eight mice were infected in each group. No significant difference in weight was observed at any time point. (PDF) [file ppat.1007511.s003.pdf]

**S4 Fig.**

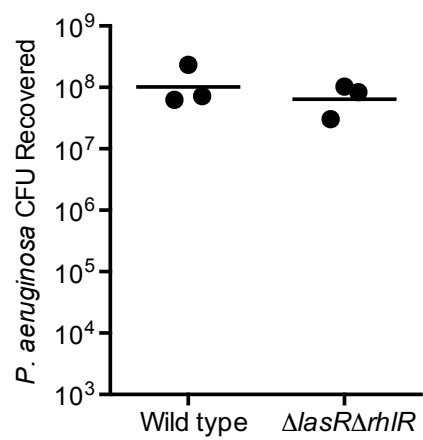

Supplement: S4 Fig — After five days of infection there was no significant difference (by student’s t-test) in the CFUs of P. aeruginosa recovered from wounds infected with wild-type and quorum sensing mutant (ΔlasRΔrhlR) P. aeruginosa. Bar indicates geometric mean of CFU counts. (PDF) [file ppat.1007511.s004.pdf]

S5 Fig.

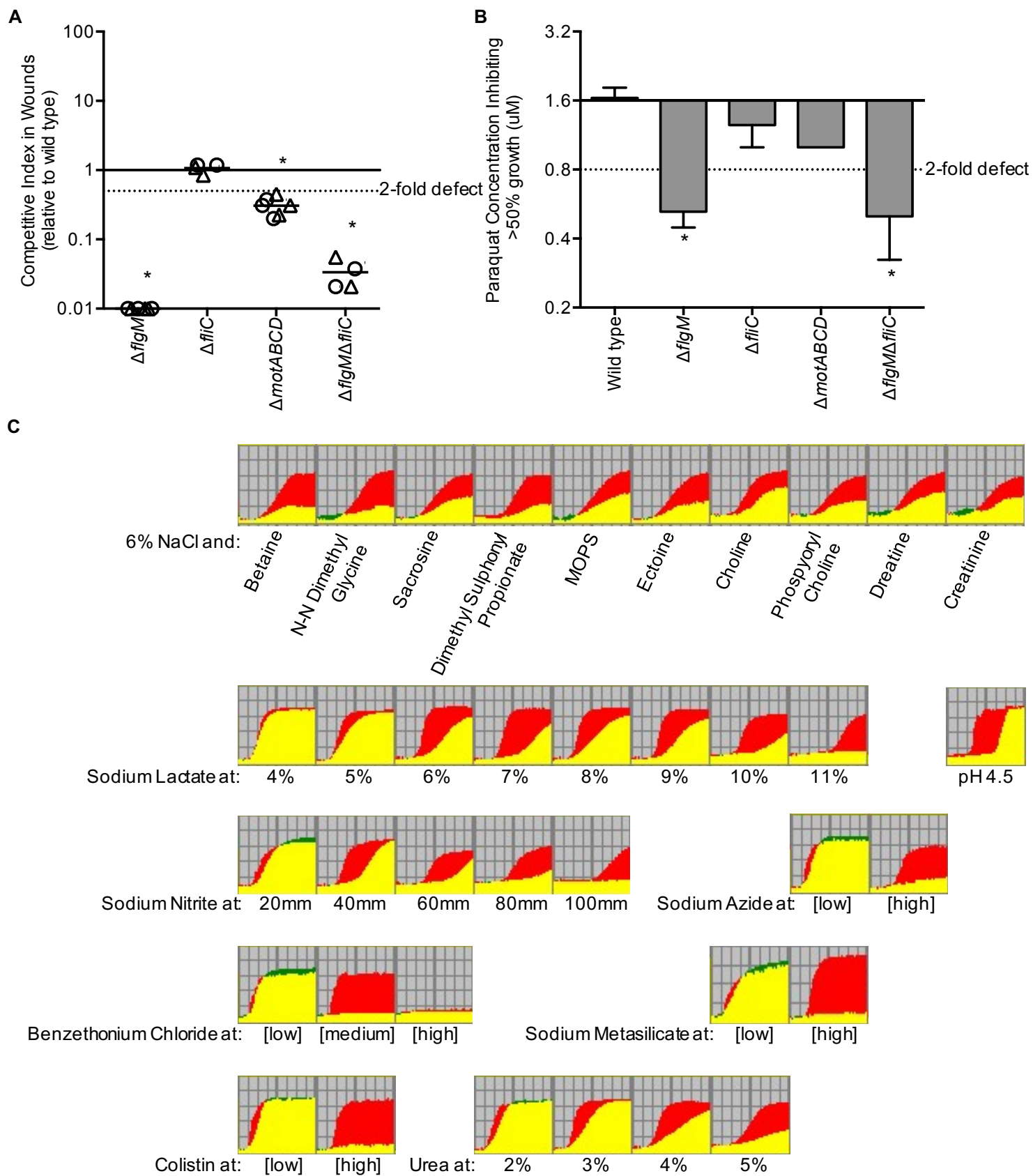

Supplement: S5 Fig — We conducted studies to investigate mechanisms explaining the severe wound fitness defect of the flgM mutant. Inactivation of flgM increases expression of flagellin (encoded by fliC), and ΔflgM P. aeruginosa produce an abnormal flagella that is non-motile. A) The wound fitness defect of ΔflgM could not be explained by its swimming motility defect, as the non-motile ΔfliC mutant exhibited no fitness defect in wounds. Likewise, the wound fitness defect of ΔflgM could not be explained by the presence of a non-motile flagella, as ΔmotABCD P. aeruginosa (which also has a non-motile flagella) exhibited near wild-type levels of wound fitness. We also tested a ΔfliC/ΔflgM double mutant, which lacks both flagella and FlgM’s regulatory actions, and found that the ΔfliC/ΔflgM mutant had a wound defect comparable to that of the ΔflgM strain. These experiments implicate some effect of FlgM (other than on motility or fliC regulation) in ΔflgM’s wound fitness defect. * indicates competitive index significantly different than 1 (p<0.05) by one sample t test. B) We tested flagellar mutants for oxidative stress sensitivity. The ΔflgM and ΔfliC/ΔflgM mutants exhibited increased paraquat sensitivity as measured by the minimum concentration of paraquat required to inhibit 50% of growth (IC50). The other non-motile mutants tested (ΔfliC and ΔmotABCD) were less sensitive to paraquat than ΔflgM and ΔfliC/ΔflgM. The x-axis is set to cross the y-axis at the value of wild-type P. aeruginosa’s paraquat IC50 for ease of visualization. C) We used Biolog Phenotypic microarrays (PMs) to identify additional stress sensitivities caused by flgM inactivation. Graphs show respiratory activity (Y-axis) as a function of time (X-axis) of wild type P. aeruginosa and the ΔflgM strain. Respiratory activity is a surrogate for growth in the indicated stress conditions. Respiratory activity of wild-type P. aeruginosa is shown by red-shaded areas; the ΔflgM strain is shown by green-shaded areas; and areas where [file ppat.1007511.s005.pdf]

**S6 Fig.**

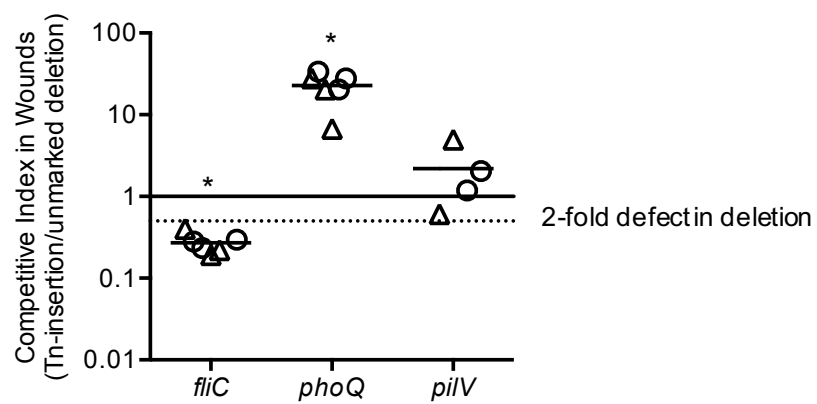

Supplement: S6 Fig — Transposon mutants were competed against unmarked deletions in the same gene. The competitive index of the transposon mutant relative to the unmarked deletion in the same gene in scabs (circle) or wound beds (triangle) five days after infection indicates no consistent trend of increased (CI>1) or decreased (CI<1) fitness due to the presence of the transposon amongst the three mutants tested. * indicates that there is a significant difference in fitness between the unmarked deletion and the transposon mutant by the 1-sample t-test. (PDF) [file ppat.1007511.s006.pdf]

A

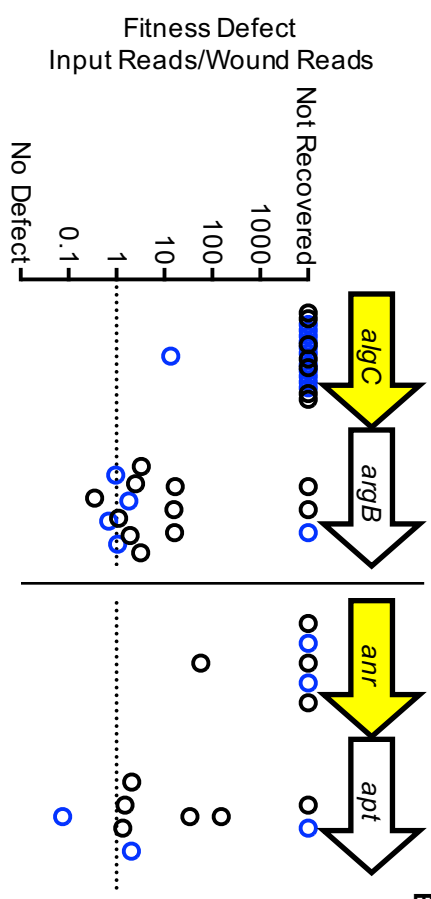

B

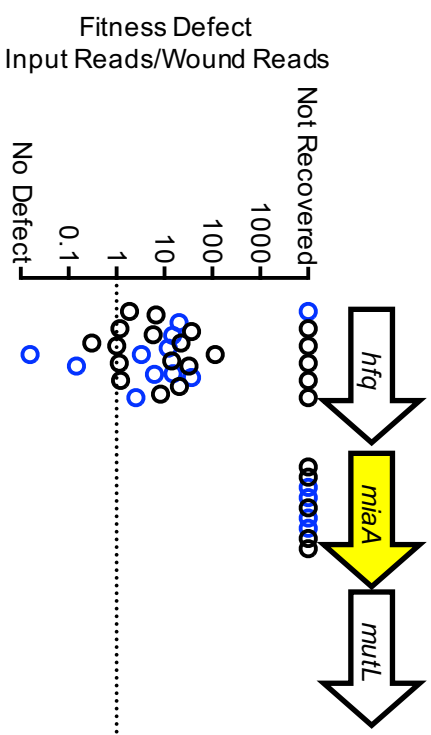

C

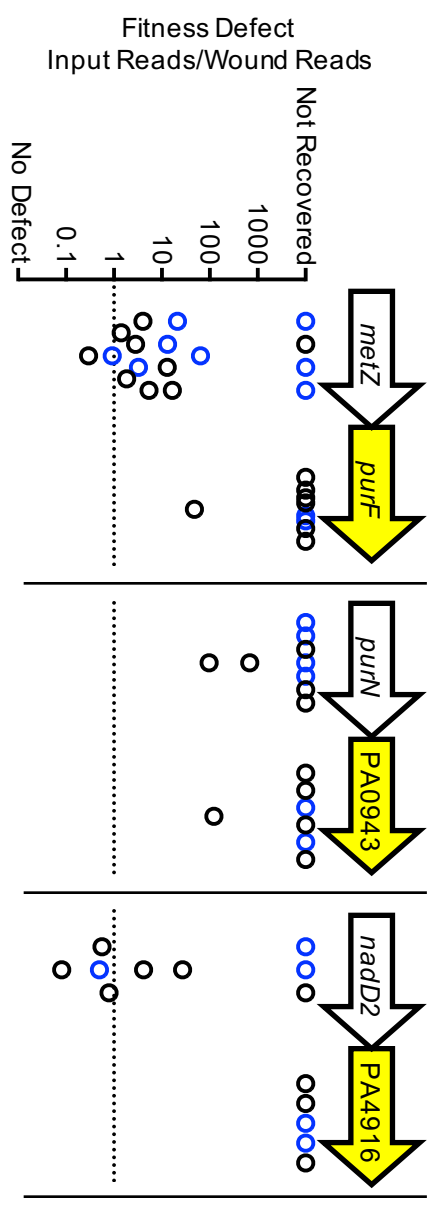

D

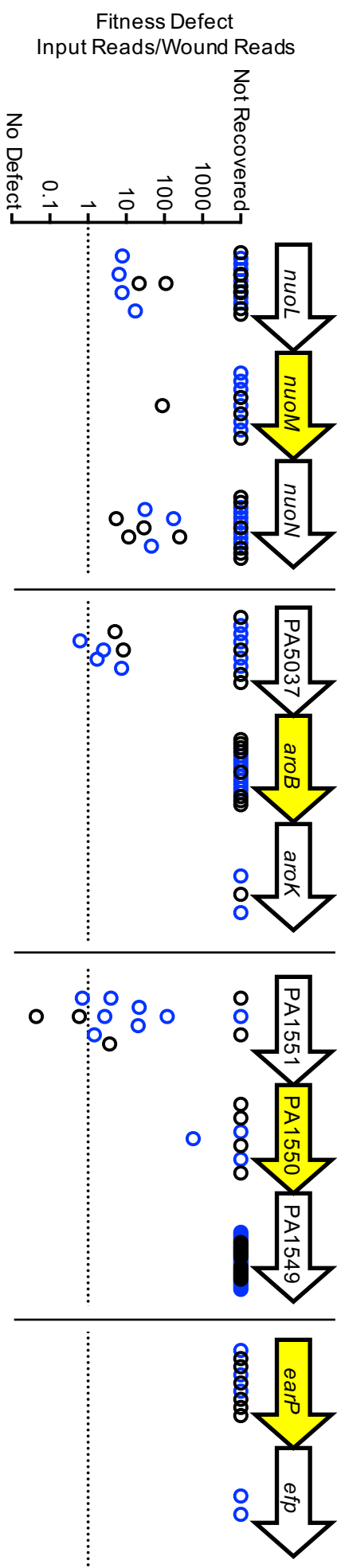

Supplement: S7 Fig — We used Tn-seq data to determine if polar effects on neighboring genes could explain the defects of mutants verified to have greater than ten-fold impairments in wound fitness (see verification experiments in Fig 4). Verified wound-defective mutants are indicated by yellow highlighting, and genes immediately up- and down-stream are not highlighted. Because the transposon we used in Tn-seq experiments is more likely to cause polar effects on downstream genes when inserted in the reverse orientation [53], we marked forward- and reverse-oriented transposon insertions with black and blue points respectively. Of the 11 verified wound-defective mutants analyzed, the fitness defects in seven (algC, anr, miaA, purF, PA0943, PA4916, and pgk) were not likely due to polar effects (see panels A-C). We cannot rule out polar effects for four verified wound-defective mutants (nuoM, aroB, PA1550, and earP) as the transposon mutant in the downstream gene was also compromised for wound fitness (see panel D). A)The transposon insertions in the genes downstream of algC, and anr (argB and apt) produced less severe wound fitness defects than the insertions in algC and anr. B) The gene downstream of miaA had no inserts. C) There was no downstream gene in the operons of purF, PA0943, PA4916, and pgk. D) The transposon mutant in the genes downstream of nuoM, aroB, PA1550, and earP were also compromised for wound fitness. However, for three of these four genes the downstream gene (nuoN, aroK, and efp) is in the same pathway as the verified wound-defective mutant (nuoM, aroB, and earP). Thus, the verified wound-defective mutant, and the transposon in the downstream gene may independently affect wound fitness. (PDF) [file ppat.1007511.s007.pdf]

S8 Fig.

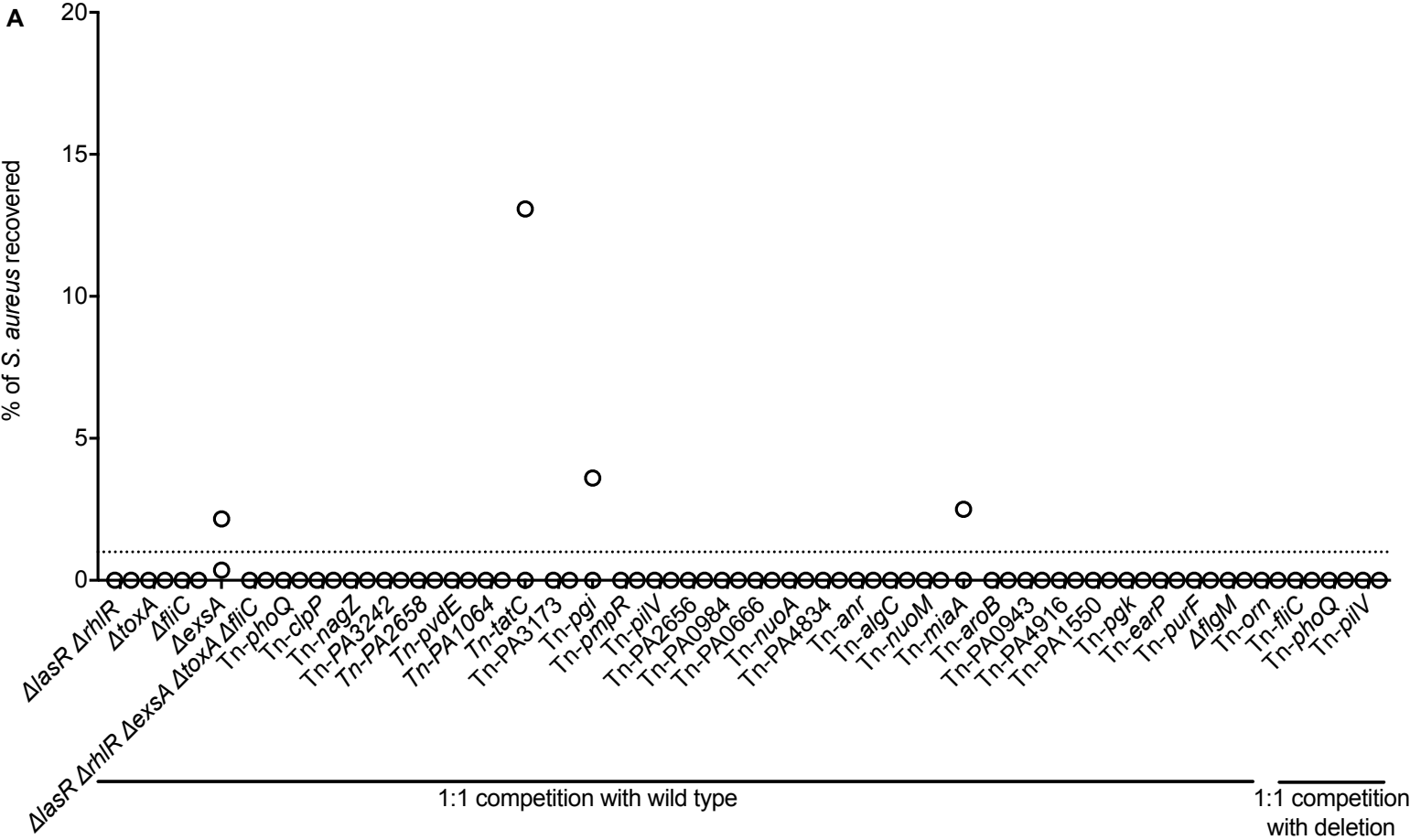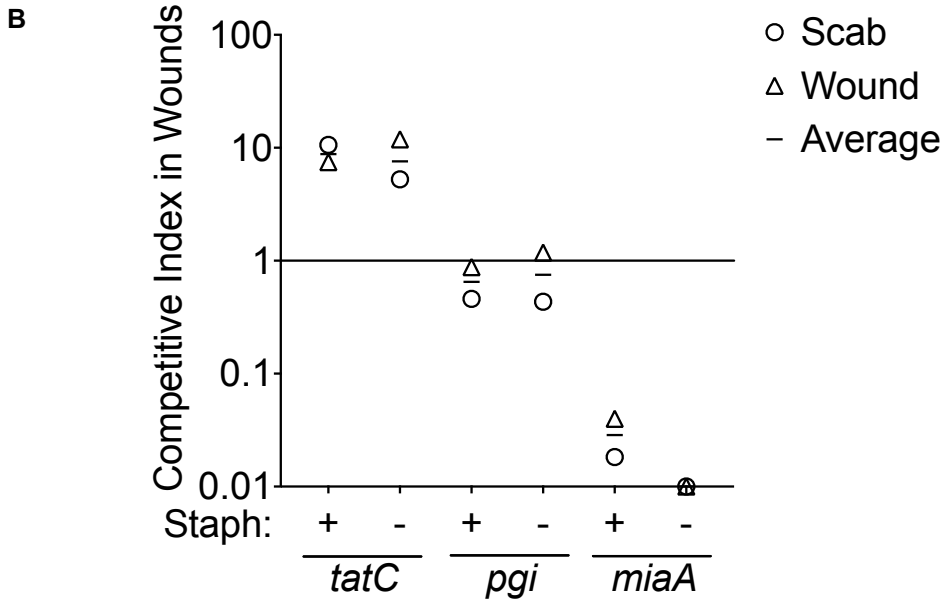

Supplement: S8 Fig — A) In the 1:1 competitions between wild-type and mutant Pseudomonas, only four wounds were contaminated with Staphylococcus. B) Competitive index information the three P. aeruginosa mutants for which one replicate experiment was contaminated with <1% Staphylococcus, and the other replicate experiment was not. The competitive index of the transposon in scabs (circle) or wound beds (triangles) for each replicate is shown. The x-axis indicates if that wound was contaminated with < 1% Staphylococcus (based on growth on Mannitol Salts Agar). (PDF) [file ppat.1007511.s008.pdf]
