# Supplementary material for: Bacterial fitness in chronic wounds appears to be mediated by the capacity for high-density growth, not virulence or biofilm functions
Source: PLoS Pathog. 2019 Mar 20;15(3):e1007511. doi: 10.1371/journal.ppat.1007511 (PMC6448920; doi:10.1371/journal.ppat.1007511)
Supplement: S2 Table — (PDF) [file ppat.1007511.s010.pdf]

**S2 Table: Competitive index of mutants in mouse wound, logarithmic culture, colony, and stationary phase**

| Strain | Mouse CI | Logarithmic CI | Colony CI | Stationary CI |
|---|---|---|---|---|
| wildtype | NT | 1.06 ± 0.27 | 1.01 ± 0.37 | 1.16 ± 0.22 |
| Tn-*phoQ* | 22.13 ± 4.33 | 1.2 ± 0.22 | 3.87 ± 1.56 | 0.91 ± 0.11 |
| Tn-*clpP* | 17.05 ± 8.49 | 1.32 ± 0.26 | 14.44 ± 3.12 | 1.1 ± 0.09 |
| Tn-*nagZ* | 16.83 ± 2.36 | 1.15 ± 0.13 | 9.06 ± 1.47 | 0.93 ± 0.08 |
| Tn-PA3242 | 16.58 ± 0 | 0.97 ± 0.11 | 3.49 ± 0.32 | 2.19 ± 0.56 |
| Tn-PA2658 | 10.93 ± 1.98 | 1.83 ± 0.14 | 4.2 ± 1.66 | 0.98 ± 0.15 |
| Tn-*pvdE* | 10.86 ± 4.98 | 1.36 ± 0.51 | 4.93 ± 1.59 | 0.89 ± 0.03 |
| Tn-PA1064 | 8.38 ± 0.29 | 5.57 ± 4.26 | 5.97 ± 2.42 | 0.67 ± 0.41 |
| Tn-*tatC* | 8.18 ± 0.87 | 0.95 ± 0.11 | 6.05 ± 2.47 | 1.59 ± 0.13 |
| Tn-PA3173 | 0.96 ± 0.68 | 1.29 ± 0.42 | 1.67 ± 0.63 | 1.13 ± 0.02 |
| Tn-*pgi* | 0.7 ± 0.07 | 0.85 ± 0.22 | 0.09 ± 0.08 | 1.62 ± 0.4 |
| Tn-*pmpR* | 0.6 ± 0.01 | 1.02 ± 0.24 | 1.33 ± 0.15 | 0.48 ± 0.02 |
| Tn-*pilV* | 0.57 ± 0.33 | 0.45 ± 0.23 | 0.14 ± 0.07 | 0.46 ± 0.28 |
| Tn-PA2656 | 0.45 ± 0.1 | 0.26 ± 0.05 | 0.8 ± 0.31 | 0.01 ± 0 |
| Tn-PA0984 | 0.33 ± 0.12 | 1.61 ± 0.36 | 1.16 ± 1.51 | 0.5 ± 0.11 |
| Tn-PA0666 | 0.16 ± 0.13 | 1.35 ± 0.41 | 6.24 ± 2.52 | 0.75 ± 0.48 |
| Tn-*nuoA* | 0.13 ± 0.08 | 1.17 ± 0.11 | 1.75 ± 0.58 | 0.24 ± 0.26 |
| Tn-PA4834 | 0.13 ± 0.08 | 1.37 ± 0.21 | 5.57 ± 1.61 | 0.41 ± 0.13 |
| Tn-*anr* | 0.05 ± 0.03 | 1.51 ± 0.28 | 0.67 ± 0.25 | 0.14 ± 0.06 |
| Tn-*algC* | 0.04 ± 0.01 | 0.26 ± 0.05 | 0.03 ± 0 | 0.1 ± 0.13 |
| Tn-*nuoM* | 0.04 ± 0.01 | 0.29 ± 0.17 | 0.01 ± 0.01 | 0.11 ± 0.1 |
| Tn-*miaA* | 0.02 ± 0.01 | 0.5 ± 0.28 | NT | 0.16 ± 0.14 |
| Tn-*aroB* | 0.02 ± 0.01 | 0.23 ± 0.1 | 0.01 ± 0 | 0.01 ± 0 |
| Tn-PA0943 | 0.01 ± 0 | 0.76 ± 0.21 | 0.01 ± 0.01 | 0.1 ± 0.01 |
| Tn-PA4916 | 0.01 ± 0 | 0.44 ± 0.1 | 0.01 ± 0 | 0.28 ± 0.2 |
| Tn-PA1550 | 0.01 ± 0 | 1.06 ± 0.13 | 0.07 ± 0.08 | 0.01 ± 0 |
| Tn-*pgk* | 0.01 ± 0 | 0.31 ± 0.26 | 0.04 ± 0.03 | 0.32 ± 0.27 |
| Tn-PA2852 | 0.01 ± 0 | 0.17 ± 0.09 | 0.01 ± 0 | 0.05 ± 0.02 |
| Tn-*purF* | 0.01 ± 0 | 0.61 ± 0.13 | 0.12 ± 0.11 | 0.22 ± 0.23 |
| Δ*flgM* | 0.01 ± 0 | 0.61 ± 0.18 | 0.06 ± 0.09 | 0.16 ± 0.03 |
| Tn-*orn* | 0.01 ± 0 | 0.24 ± 0.08 | NT | 0.08 ± 0.03 |
