# Supplementary material for: Bacterial fitness in chronic wounds appears to be mediated by the capacity for high-density growth, not virulence or biofilm functions
Source: PLoS Pathog. 2019 Mar 20;15(3):e1007511. doi: 10.1371/journal.ppat.1007511 (PMC6448920; doi:10.1371/journal.ppat.1007511)
Supplement: S4 Table — (PDF) [file ppat.1007511.s012.pdf]

S4 Table: Strains used in this study

Strain	Genotype	Source
Wildtype	mPAO1	[30]
Δ*lasR*Δ*rhlR*	mPAO1Δ*lasR*Δ*rhlR*	[54]
Δ*exsA*	mPAO1Δ*exsA*	This Study
Δ*toxA*	mPAO1Δ*toxA*	This Study
Δ*fliC*	mPAO1Δ*fliC*	This Study
Δ*lasR*Δ*rhlR*Δ*exsA*Δ*toxA*Δ*fliC*	mPAO1Δ*lasR*Δ*rhlR*Δ*exsA*Δ*toxA*Δ*fliC*	This Study
Δ*pslD*	mPAO1Δ*pslD*	[55]
Δ*wspF*Δ*pel*Δ*psl*	mPAO1Δ*wspF*Δ*pelF*Δ*pslD*	This Study
Δ*wspF*	mPAO1Δ*wspF*	[27]
Tn-phoQ	*phoQ*-F10::ISphoA/hah	[30]
Tn-clpP	*clpP*-A05::ISlacZ/hah	[30]
Tn-nagZ	*nagZ*-B02::ISlacZ/hah	[30]
Tn-PA3242	PA3242-E09::ISlacZ/hah	[30]
Tn-PA2658	PA2658-F03::ISphoA/hah	[30]
Tn-*pvdE*	*pvdE*-D05::ISSupp/hah	[30]
Tn-PA1064	PA1064-H08::ISphoA/hah	[30]
Tn-*tatC*	*tatC*-A10::ISlacZ/hah	[30]
Tn-PA3173	PA3173-A04::ISlacZ/hah	[30]
Tn-*pgi*	*pgi*-F10::ISphoA/hah	[30]
Tn-*pmpR*	*pmpR*-C03::ISlacZ/hah	[30]
Tn-*pilV*	*pilV*-E01::ISphoA/hah	[30]
Tn-PA2656	PA2656-C11::ISphoA/hah	[30]
Tn-PA0984	PA0984-B05::ISphoA/hah	[30]
Tn-PA0666	PA0666-B07::ISphoA/hah	[30]
Tn-*nuoA*	*nuoA*-D12::ISphoA/hah	[30]
Tn-PA4834	PA4834-H06::ISlacZ/hah	[30]
Tn-*anr*	*anr*-E05::ISlacZ/hah	[30]
Tn-*algC*	*algC*-D07::ISphoA/hah	[30]
Tn-*nuoM*	*nuoM*-B03::ISphoA/hah	[30]
Tn-*miaA*	*miaA*-D01::ISphoA/hah	[30]
Tn-*aroB*	*aroB*-E11::ISlacZ/hah	[30]
Tn-PA0943	PA0943-A04::ISlacZ/hah	[30]
Tn-PA4916	PA4916-F01::ISphoA/hah	[30]
Tn-PA1550	PA1550-F09::ISphoA/hah	[30]
Tn-*pgk*	*pgk*-C01::ISphoA/hah	[30]
Tn-PA2852	PA2852-E12::ISphoA/hah	[30]
Tn-*purF*	*purF*-C09::ISphoA/hah	[30]
Δ*flgM*	mPAO1Δ*flgM*	This Study
Tn-*orn*	*orn*-G11::ISlacZ/hah	[30]
Δ*motABCD*	mPAO1Δ*motABCD*	This Study
Δ*flgM*Δ*fliC*	mPAO1Δ*flgM*Δ*fliC*	This Study
Δ*phoQ*	mPAO1Δ*phoQ*	This Study
Tn-*fliC*	*fliC*-G10::ISphoA/hah	[30]
Δ*pilV*	mPAO1Δ*pilV*	This Study
